# Pre-existing activation states shape functional heterogeneity of human Vγ9Vδ2 T cells

**DOI:** 10.3389/fimmu.2026.1696469

**Published:** 2026-02-16

**Authors:** Anna Vyborova, Laia Gasull-Celades, Peter Brazda, Alberto Miranda Bedate, Froso Karaiskaki, Jasper Sanders, Anke Janssen, Trudy Straetemans, Dennis X. Beringer, Zsolt Sebestyen, Jürgen Kuball

**Affiliations:** 1 Center for Translational Immunology, University Medical Center Utrecht, Utrecht University, Utrecht, Netherlands; 2 Department of Hematology, University Medical Center Utrecht, Utrecht University, Utrecht, Netherlands

**Keywords:** ATMP, cancer immune cell therapy, gdT cell, heterogeneity, transcriptomics

## Abstract

γδ T cells gain increasing attention as carriers for tumor-targeting constructs in therapeutic contexts. However, the failure to fully account for the diversity within the subset has impeded its clinical use so far. We investigated the heterogeneity of the Vγ9Vδ2 T-cell compartment by profiling the function and gene expression of single-cell clones expanded *in vitro* using the rapid expansion protocol (REP), which involves repeated stimulation with interleukin (IL)-2 and IL-15. Generally known to enhance the type 1 effector program in the γδ T cells, these culture conditions polarized only a proportion of the adult peripheral blood-derived clones toward “classic” type 1 effectors marked by high interferon gamma (IFN-γ) release (HIR). Unexpectedly, a substantial fraction of the clones exhibited a low-IFN-γ-releasing (LIR) profile and instead activated a type 2-like effector program, marked by IL-4 and IL-5 secretion and expression of the transcription factor GATA3. In line with this functional dichotomy, we observed coordinated transcriptional programs linking effector function to genes associated with T-cell activation, proliferation, and cytokine production. HIR clones exhibited a more activated transcriptional profile in culture compared with LIR clones. Importantly, projection of HIR and LIR gene signatures onto *ex vivo* single-cell transcriptomic data demonstrated that these effector states are already present *in vivo* as part of a continuous activation landscape within nonexpanded Vγ9Vδ2 T cells, with LIR-like states predominating in cord blood and remaining prevalent in adult peripheral blood. These findings indicate that the functional divergence observed after *in vitro* expansion reflects stabilization and amplification of preexisting activation states rather than culture-induced polarization. Analysis of the Vγ9Vδ2 T-cell receptor repertoire further suggested that intrinsic signaling features may modulate, but do not dictate, effector differentiation within this activation continuum. In summary, our data indicate that effector differentiation of Vγ9Vδ2 T cells is dominated by a preexisting LIR-like activation state, a finding with major implications for current γδ T-cell-based cancer immunotherapy strategies that rely on *in vivo* stimulation or *ex vivo* engineering.

## Introduction

The past few years have witnessed a rapid expansion in the range of targets and effector designs for adoptive cell therapies (ACTs), catalyzed by unprecedented therapeutic successes in using chimeric antigen receptor (CAR)-engineered autologous αβ T cells in heavily pretreated hematological malignancies (1). The overall rates of durable responses without the need for consolidative treatment, however, remain dismally low (2), and the responses vary significantly depending on patient features and the characteristics of the cellular product (3). In addition to the cell engineering strategy, the phenotypic and functional heterogeneity of the carrier cells has been a key determinant of ACT outcomes in both transgenic T-cell receptor (TCR) (tTCR) and CAR T trials, as it underlies the magnitude of T-cell expansion, its quality—assessed by specific cytokine production and lysis of target cells—as well as the duration of response, which often depends on the persistence of the infused product. Specifically, T-cell stemness vs. effectorness, activation vs. exhaustion state, CD4:CD8 balance, and polyfunctionality have been shown to play a decisive role in patient outcomes (3, 4). Recently, preserved type 2 functionality in CAR T infusion products has been linked to improved long-term outcomes (5), underscoring the importance of balancing effector identities in ACT products to enhance efficacy.

Simultaneously, alternative allogeneic carriers with no need for Human Leukocyte Antigen (HLA) matching, such as natural killer (NK) cells, are being actively explored in an attempt to mitigate the manufacturing costs of autologous CAR T cells, to eliminate the factor of significant patient-to-patient heterogeneity, and, in addition, to harness the innate recognition mechanisms that would remain effective against CAR antigen escape variants (6). Similar to the CAR T trials, peak expansion, persistence, and polyfunctionality of the CAR NK cells predicted the outcomes of the allogeneic CAR NK treatments (7). An alternative subset is the population of human γδ T cells, which comprises 1%–10% of CD3^+^ cells in human peripheral blood (PB) and functions largely in an HLA-unrestricted manner, making them a promising off-the-shelf allogeneic carrier for tTCRs, CARs, or other tumor-targeting constructs (8). Their natural ligands are signals of cellular stress, which do not undergo antigen processing (9), while their recognition and effector machineries feature aspects of both adaptive and innate immunity.

Vγ9Vδ2 T-cell neighbor NK cells transcriptionally (10, 11) and respond polyclonally via a semi-invariant Vγ9Vδ2TCR to changes in surface BTN2A1/BTN3A1/BTN3A2 complex upon accumulation of stress-induced intracellular pyrophosphate antigens (12), thus forming an innate axis within the landscape of γδ T cells. Both effector Vδ1 and Vγ9Vδ2 T cells exhibit potent TCR-mediated cytotoxicity and type 1 cytokine responses *in vitro* (13). Furthermore, preclinical immunotherapeutic models highlight the natural cytotoxicity receptor-mediated cytotoxicity (14) and antigen-presenting properties (15) as intriguing features with the potential to enhance the TCR/CAR-mediated effector functions and achieve antigen spreading. The outcomes of the early trials utilizing bulk autologous expanded Vγ9Vδ2 T cells had been discouraging, with virtually no durable responses observed (16). As with conventional αβ T-cell-based ACTs, the phenotypic and functional heterogeneity within the γδ subset proved to be key (17–19); therefore, the focus has shifted to nonengineered allogeneic Vδ1 cells (14) and CAR-engineered allogeneic Vδ1 (20) and Vδ2 (21) cells tailored to favorable phenotypes and functionalities, either through specific culture conditions (14) or, alternatively, through selection of sub-subpopulations (8, 21, 22).

Here, we performed functional profiling and transcriptomic analysis of the Vγ9Vδ2 single T-cell clones isolated from the PB of healthy donors and expanded using a rapid expansion protocol (REP) (23), in which cells were repeatedly exposed to IL-2 and IL-15. Previously reported to enhance the type 1 effector program in the γδ PBLs (24), these culture conditions indeed resulted in a strong type 1 signature, particularly reflected by IFN-γ release, albeit only in a fraction of clones. High IFN-γ-releasing clones were polyfunctional while maintaining high proliferative capacity, indicating their potential utility for ACT applications. A significant proportion of clones, however, activated a type 2-like effector program under the same culture conditions, manifested in IL-5 secretion. By integrating clonal transcriptomic and TCR repertoire analyses with the projection of HIR and LIR gene signatures onto *ex vivo* single-cell transcriptomic data, we demonstrate that these effector states are already present *in vivo* as part of a continuous activation landscape. Together, these findings indicate that functional divergence after *in vitro* expansion reflects stabilization and amplification of preexisting, cell-intrinsic activation states, rather than culture-induced polarization, findings directly relevant to γδ T-cell-based immunotherapy.

## Materials and methods

### Cell culture, flow cytometry, and functional testing

PB from anonymous healthy donors was purchased from Sanquin, the Dutch blood bank. All single-cell FACS sorts were performed on ARIAII (BD) using monoclonal antibody (mAb) Vδ2-FITC clone B6 (Biolegend Cat. No. 2257030, San Diego, CA). Adherent cell lines (HEK293FT, SSC9) were cultured in DMEM (Gibco^TM^, Waltham, MA, USA), while the cell lines Daudi, HL60, and RPMI8226 were cultured in RPMI (Gibco), with both media supplemented with 10% FCS and 1% penicillin/streptomycin. Vγ9Vδ2 T-cell clones were expanded following a rapid expansion protocol (REP). Briefly, on day 0, clones were cultured in RPMI-1640 medium (Gibco) containing 10% human serum, 1% penicillin/streptomycin (100 U/mL, 100 μg/mL), and β-mercaptoethanol (50 μM, Merck & Co., Inc. Rahway, New Jersey, USA), as well as irradiated feeders consisting of Peripheral blood mononuclear cells (PBMCs) (1 × 10^6^ cells/mL, 3,500 cGy), LCL-EBV-transformed B cells (0.1 × 10^6^ cells/mL, 8,000 cGy), and Daudi cells (0.1 × 10^6^ cells/mL, 8,000 cGy) as previously described (25), supplemented with IL-2 (50 U/mL, R&D Systems), IL-15 (5 ng/mL, R&D Systems, Minneapolis, MN, USA), and PHA (1 μg/mL, Sigma-Aldrich, St. Louis, MO, USA). Every 3 days, the media was refreshed, and IL-2 (50 U/mL) and IL-15 were added (5 ng/mL). The different REP culturing conditions, Th1- or Th2-polarizing, follow the same protocol, in which Th1 is the same as described above, and Th2 differs in the addition of IL-4 (20 ng/mL, Miltenyi Biotec, Bergisch Gladbach, Rhineland, Germany) and IL-13 (20 ng/mL, Invitrogen, Carlsbad, CA, USA), while IL-15 is also added. The single-cell sorting of the Vδ2 T-cell clones, cell culture, and functional testing, including coincubation with tumor cell lines, IFN-γ ELISA, and Luminex assay, were performed as described previously (26).

### RNA isolation and sequencing

RNA isolation was performed using the RNeasy Mini Kit (Qiagen, Hilden, Germany) according to the manufacturer’s instructions. The quantity and quality of RNA were assessed with Bioanalyzer (Agilent, Santa Clara, California, USA), applying the minichip analysis. A minimal RNA integrity number (RIN) of 7 in any sample was considered suitable for sequencing. RNA libraries were obtained with TruSeq Stranded mRNA Library Prep for NeoPrep (Illumina, San Diego, California, USA), and sequenced on an Illumina NextSeq 500 sequencer with a High-Output Kit (Illumina) in a single-end 1 × 75 bp format (USEQ, Utrecht, The Netherlands). The average number of total reads per sample was approximately 14*e*06.

### RNAseq analysis

FastQ files were first subjected to quality control assessment using the FastQC tool to evaluate read quality and identify potential technical issues. High-quality reads from each sample were then aligned to the human reference genome using the STAR aligner, producing the raw counts per gene. Raw read counts were filtered to retain genes with at least 1 count per million (CPM) in a minimum of three samples, and gene expression distributions were normalized using the trimmed mean of *M*-values (TMM) method implemented in edgeR. DEG analysis was performed using the DESeq2 R package under standard parameters. Due to patient privacy considerations, The raw sequencing data are available in the NCBI Sequence Read Archive (BioProject ID PRJNA1418759)

### Pathway analysis

#### GSEA and ssGSEA

For the group-level Gene Set Enrichment Analysis (GSEA), we used the software downloaded from the GSEA Website (https://www.gsea-msigdb.org/gsea/index.jsp) under the default settings. Cytoscape (27) was used for visualizing the gene set networks.

Single-cell GSEA (ssGSEA) was performed using the Gene Set Variation Analysis (GSVA) package (28) in RStudio.

### TCR sequencing

TCR sequencing of the expanded primary T-cell clones, as well as high-throughput sequencing (HTS) of the TCRδ chain, was performed as previously described (26). The International Immunogenetics Information System (IMGT) V-QUEST tool (29, 30) was used for CDR3 mapping and annotation. We therefore report the CDR3 length in accordance with the IMGT nomenclature, with the conserved cysteines and phenylalanines not included.

### Single-cell RNA-seq analysis and signature projection

Publicly available single-cell RNA-seq data (GSE149356) from human γδ T cells were obtained and processed in *Seurat* (v4.0.1). Cells expressing both TRGV9 and TRDV2 were subsetted to isolate Vγ9Vδ2 T cells. To assess inflammatory transcriptional states, two predefined gene sets were used: a high-inflammatory response (HIR) signature and a low-inflammatory response (LIR) signature, derived from differentially expressed genes in clonally expanded Vγ9Vδ2 T cells. Differentially expressed genes were identified by first subsetting for protein-coding genes, followed by applying thresholds of adjusted *p*-value < 0.05 and absolute log_2_ fold change ≥ 1. The Seurat function *AddModuleScore*() was used to compute enrichment scores for each gene set at the single-cell level. For classification, each cell was assigned to either the HIR or LIR category using a mutually exclusive, threshold-free strategy based on which module score was greater. This binary classification was used to compute the relative proportions of HIR and LIR cells within each donor. Fisher’s exact test was performed to evaluate whether the distribution of HIR and LIR cells was significantly different between cord blood and PB samples. Module score distributions were visualized using violin plots (*VlnPlot*), and single-cell-level enrichment was visualized via heatmaps using the *ComplexHeatmap* R package. Cells were grouped by donor identity. All statistical analysis and visualization were performed in *R* (v4.0.5).

## Results

### IFN-γ signature reflects Vγ9Vδ2 T-cell polyfunctionality

**Figure 1:**
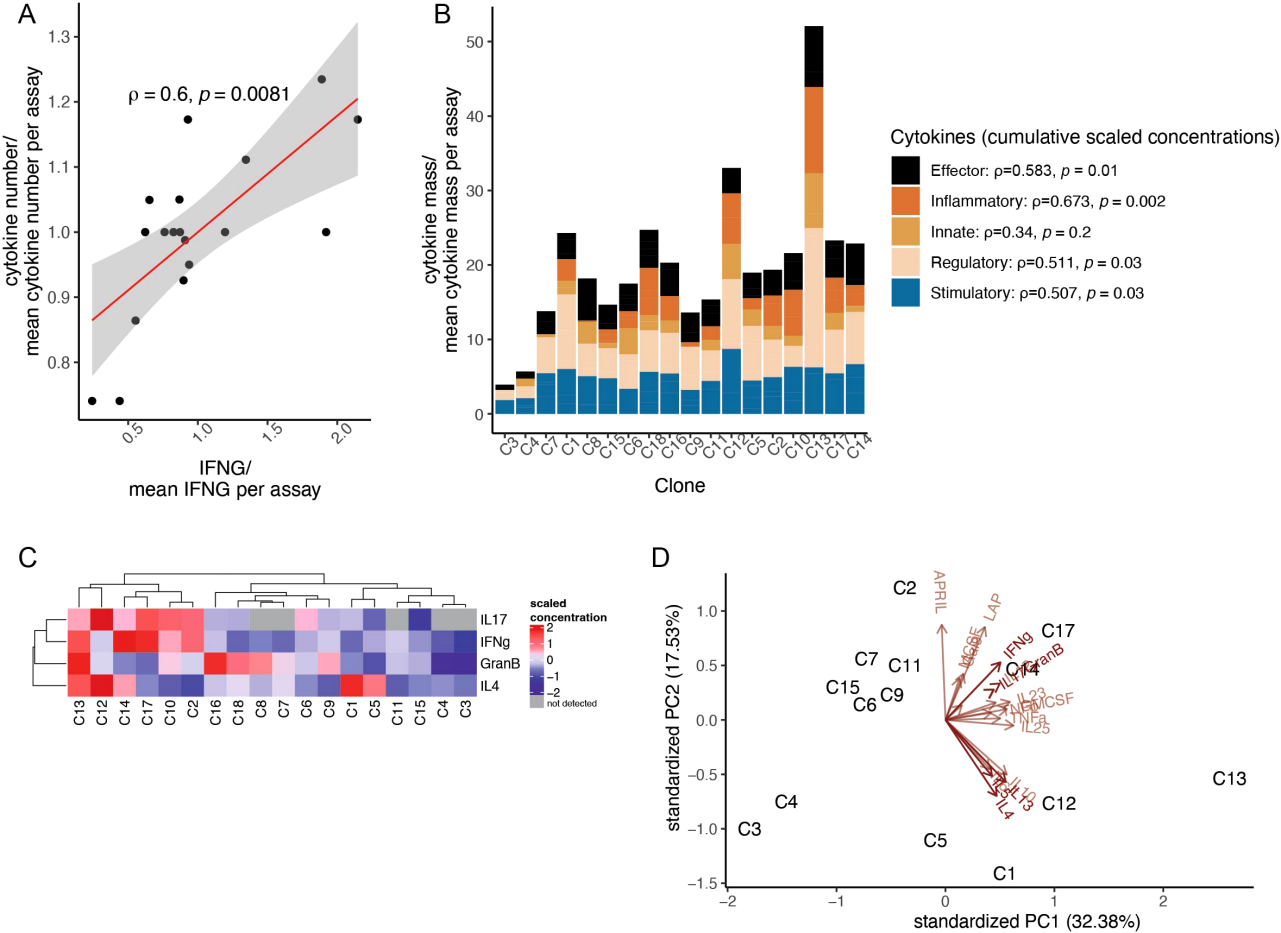
High IFN-γ release marks polyfunctional Vγ9Vδ2 T-cell clones. **(A)** Number of secreted cytokines scaled per assay and plotted against the magnitude of IFN-γ release (clones from donor C; Daudi cells used as targets). **(B)** Secreted cytokine mass calculated as the sum of scaled cytokine concentrations in the assay supernatants. Clones are ordered by increasing IFN-γ release (clones from donor C; Daudi cells as targets). Cytokines were classified as effector (GranB, IFN-γ, TNF-α, TNF-β), stimulatory (GMCSF, MCSF, IL-2, IL-5, IL-15, IL-7, APRIL), regulatory (LAP, IL-4, IL-10, IL-13, Gal9, FASL), inflammatory (IL-17, IL-17F, IL-25, IL-23), and innate (IL-6, IL-12, IL-18). The Spearman correlation between IFN-γ release and the cumulative scaled concentrations of other classes of cytokines is shown in the legend. **(C)** Heatmap showing scaled concentration of a selection of cytokines (Daudi cell line as target). **(D)** Principal component analysis (PCA) of the cytokine data shown in **(B)**.

In our previous analysis, we found that sorting single Vγ9Vδ2 T cells from healthy donors and expanding them using REP gave rise to low- and high-IFN-γ-releasing (LIR and HIR) clones, as measured by tumor-induced IFN-γ production (26). The magnitude of IFN-γ release marks polyfunctional (i.e., exhibiting multiple effector functions) αβ T cells with high antitumor potency (4); however, it remained unclear whether consistent patterns applied to γδ T cells. To assess more broadly the secretory function of the LIRγδ and the HIRγδ clones after REP, we measured tumor-induced release of an additional array of 22 cytokines in the supernatants of the clones from a single donor in the aforementioned study (26) (donor C). IFN-γ protein production levels correlated both with the overall number of secreted cytokines (**Figure 1A**) and with the secreted “cytokine mass”, expressed as the total protein amount of all assessed analytes (**Figure 1B**), thus acting as an indirect measure of T-cell polyfunctionality. Notably, the release of not only type 1 effector molecules, such as tumor necrosis factor (TNF)-α, correlated with the IFN-γ readout. Correlations were also seen with stimulatory cytokines IL-2 and GM-CSF, regulatory cytokines such as galectin 9 (Gal9), and inflammatory cytokines (**Figure 1B**). Projected onto this apparent continuum of what could be seen as general cell fitness or “effectorness”, one could define subgroups of clones coproducing archetypal type 2, type 3 cytokines, and cytolytic mediators (**Figure 1C**) in a seemingly clone-intrinsic rather than tumor-selective manner, as responses to the cell lines of different tissue origin were highly correlated (**Supplementary Figure S1A**). Although secretion of IL-17 under these conditions was detectable in 72% of clones and correlated with IFN-γ production, the absolute concentration values were below 20 pg/ml (**Supplementary Figure S1B**). Unsupervised analysis of the cytokine secretion pattern discerned a cluster of clones (clones C1, C5, C12, and C13), separated by the second principal component, which was polarized toward IL-4, IL-5, and IL-13 production as opposed to the bulk of the type 1-programmed (Th1)/cytotoxic lymphocyte (CTL)-type clones (**Figure 1D**). Thus, alongside a gradient of type 1/cytotoxic effector functionalities, a subgroup of REP-expanded clones showed a type 2 effector bias in their antitumor cytokine production, while no appreciable skewing toward a type 3 response occurred in the REP culture.

### Global transcriptomic profiling of clonal effector states

**Figure 2:**
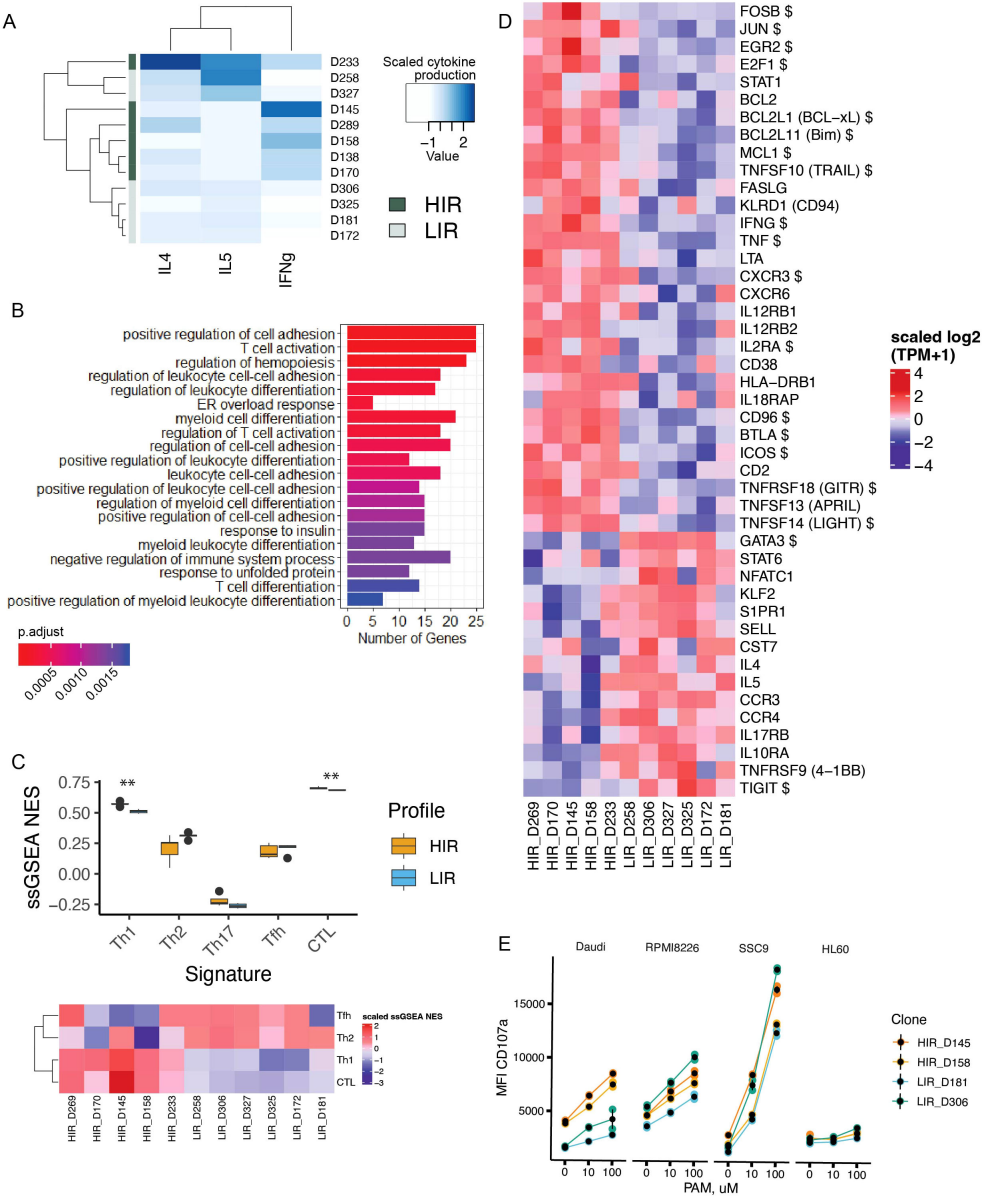
Transcriptional signature underlying HIR/LIR dichotomy in the donor D. **(A)** Heatmap of cytokine concentration secreted by clones from donor D upon coincubation with HEK293FT cells. The HIR/LIR signature was determined based on the magnitude of IFN-γ release. **(B)** Gene Ontology (GO) biological processes annotation of the DEGs in HIR cells. **(C)** Top: Single-sample GSEA normalized enrichment scores (NES) of curated gene signatures defining T helper (Th) and cytotoxic T lymphocyte (CTL) effector programs. Bottom: Values as in **(B)**, z-score normalized and plotted for individual clones. **(D)** Heatmap showing z-score-normalized expression of several immune genes of interest. DEGs are marked with a dollar sign ($), whereas, for other genes depicted in the heatmap, a lenient threshold of *p* < 0.2 for a single-gene comparison between HIR and LIR groups was used for exploratory purposes. **(E)** CD107a degranulation assay performed with a selection of clones (two HIR and two LIR) against a panel of four target tumor cell lines. ** means a p<0.01.

In order to further explore the biology behind the IFN-γ release potential of the cultured clones, we performed an additional round of clone isolation, expansion, and functional testing as previously described (26) (donor D). Among the clones isolated from this donor, we again observed a gradient in IFN-γ response to tumor cell lines, which we partitioned into HIR and LIR clusters using the k-means algorithm (*k* = 2) (**Supplementary Figure S2A**). Consequently, the dichotomy based on production of IFN-γ vs. IL-4 and IL-5, as detected in the PC2 of the cytokine data from donor C, was confirmed in donor D (**Figure 2A**). The profiles proved to be consistent over the course of time, as we observed no major crossover between the HIR and LIR clusters for clones that were tested repeatedly after another REP round (**Supplementary Figure S2B**). We further used a selection of clones from this experiment (REP3, *n* = 11) to perform bulk RNA sequencing of the expanded HIR and LIR clones in the resting state in culture.

To explore the broad transcriptional activity in relation to the effector profiles, we used the curated hallmark datasets from the MSigDB collection (31), together with the blood transcription modules (BTMs), which provide a framework of functionally related gene sets for the study of immune cell subsets in the PB (32), to perform group-level GSEA. In parallel, we performed the DEG analysis, which revealed 513 DEGs between HIRs and LIRs (**Supplementary Table S1**). The HIRs stood out primarily as enriched in cell cycle genes, E2F targets, and DNA repair genes, emphasizing their proliferative activity (**Supplementary Figure S2C**, left). When we used the cell counts during *in vitro* culturing as an approximation of their proliferative potential, it appeared that HIRs indeed expanded more rapidly in the first weeks after isolation (**Supplementary Figure S2D**). LIR clones showed a delay in peak density in culture, with subsequent gradual loss of proliferative capacity in both HIR and LIR groups. Other pathway hubs enriched in HIR cells included IFN-γ and TNF-α signaling and T-cell activation (**Supplementary Figure S2C**, middle and right panels). Gene Ontology (GO) enrichment analysis of the DEGs echoed the GSEA performed on the whole dataset, retrieving biological processes such as T-cell activation, differentiation, and adhesion for the HIR cells (**Figure 2B**). LIR cells showed no significantly enriched hallmarks or BTMs, while GO annotation of the DEGs revealed terms related to RNA, DNA, and noncoding RNA metabolism (**Supplementary Figure S2E**).

### Transcriptomic signatures of effector heterogeneity

We then studied the effector functions of the clones at the transcriptomic level. The amount of stored IFN-γ, IL-4, and IL-5 mRNA correlated with the cytokine protein concentration measured upon coincubation with Daudi or HEK293FT cells (**Supplementary Figure S2F**), suggesting that these effector functions were “preprogrammed” and clone-intrinsic. Indeed, when we projected the curated gene sets defining the canonical CTL/Th signatures (33, 34) onto the transcriptomes of the expanded clones using single-sample GSEA (ssGSEA), all clones showed an overall CTL > Th1 > Th2 > Tfh > Th17 enrichment pattern; however, the Th1 signature was differentially enriched in HIR cells, while Th2/Tfh signatures were more prominent in the LIRs (**Figure 2C**). Gene-level analysis of the archetypal effector programs in HIR and LIR cells (see **Figure 2D** for selected genes) showed comparable expression of T-bet (*TBX21*) and Eomesodermin (*EOMES*), known as the principal drivers of the type 1 and CTL effector programs in various lymphoid subsets (35, 36) (data not shown). In contrast, other type 1-associated transcripts, such as *IFNG*, *TNF* (TNF-α), *LTA* (TNF-β), *CXCR3*, *IL12R*, and *IL18R* subunits, were clearly enriched in the HIR cluster. Neither perforin nor granzyme mRNA expression segregated clearly with HIR or LIR clusters (data not shown), although HIR clones showed moderately higher granzyme B protein secretion upon tumor challenge (**Supplementary Figure S2G**). Other cytotoxicity-associated genes distinguished HIR and LIR clusters, with HIR cells expressing higher levels of death ligands *TNFSF10* (TRAIL) and *FASLG* (FAS ligand), whereas LIR cells tended to express more cystatin F (*CST7*). To test the antitumor cytotoxicity potential of the HIR vs. LIR clusters, we performed a CD107 degranulation assay with a selection of clones (*n* = 4), in which HIR clones showed a greater cytotoxic response to the Daudi target cell line (**Figure 2E**). However, unlike cytokine production, the cytotoxic response proved to be cell line-specific, suggesting, together with the heterogeneity in expression of cytotoxicity genes, that the HIR vs. LIR clones might deploy alternative cytotoxicity mechanisms and be effective against different targets.

Most intriguingly, and likely explanatory of the observed IL-4 and IL-5 release upon the *in vitro* challenge, LIR cells expressed higher levels of the Th2-associated “master regulator” *GATA3*, as well as the transcription factors *STAT6* and *NFATC1* at baseline (**Figure 2D**). This “type 2-like” profile was additionally corroborated by the higher levels of type 2-associated surface receptors C-C motif chemokine receptor 3 (*CCR3*) and *CCR4* and expression of *IL17RB* (IL-25 receptor) on LIR clones.

Expanding the functional profiling of the clones beyond the canonical effector trajectories, we noted differential expression of the two TNF superfamily members *TNFSF13* (APRIL) and *TNFSF14* (LIGHT) in HIR clones. Interestingly, we found *APRIL* to be the most significantly coexpressed cytokine alongside *IFNG* at the mRNA level (Spearman’s *ρ* = 0.94, *p* = 2.2*e*−16), an observation that was confirmed by coexpression of these two cytokines at the protein level in the donor C (**Figure 1D**).

### Activation state as a determinant of functional plasticity

Notably, we observed coexpression patterns that linked the effector profiles of the clones to their activation state. The top genes most strongly coexpressed with IFN-γ included IL-2/IL-15 target genes associated with cell proliferation and survival, such as antiapoptotic proteins Bcl-2, Bcl-xL, and Mcl-1; activator protein-1 (AP-1) complex components c-Jun and FosB; and the IL-2 receptor α chain (CD25), reflecting the generic process of T-cell activation. A range of DEGs revealed differential patterns of coactivation and coinhibition of the TCR signaling pathway. Specifically, HIR cells expressed *ICOS*, *CD2*, and *TNFRSF18* (GITR), while LIR clones showed higher *TNFRSF9* (4-1BB) expression. Of the regulatory axes, *BTLA* and *CD96* were differentially upregulated in HIR clones, whereas LIR clones exhibited higher expression of *TIGIT*. *CD69*, an early readout for T-cell activation across multiple axes, did not differentiate between HIR and LIR clones (data not shown). In contrast to the HIR clones, which expressed late activation markers *IL2RA*, *CD38*, and *HLA-DR*, LIR clones maintained high levels of the naïve markers L-selectin (*SELL*) and sphingosine-1-phosphate receptor 1 (*S1PR1*), together with their controlling transcription factor *KLF2*. The last three genes are downregulated in activated cells and are associated with maintaining T-cell quiescence (37). Together, these findings suggest that LIR clones remain in a less activated state in culture.

### TCR signaling strength modulates effector divergence

**Figure 3:**
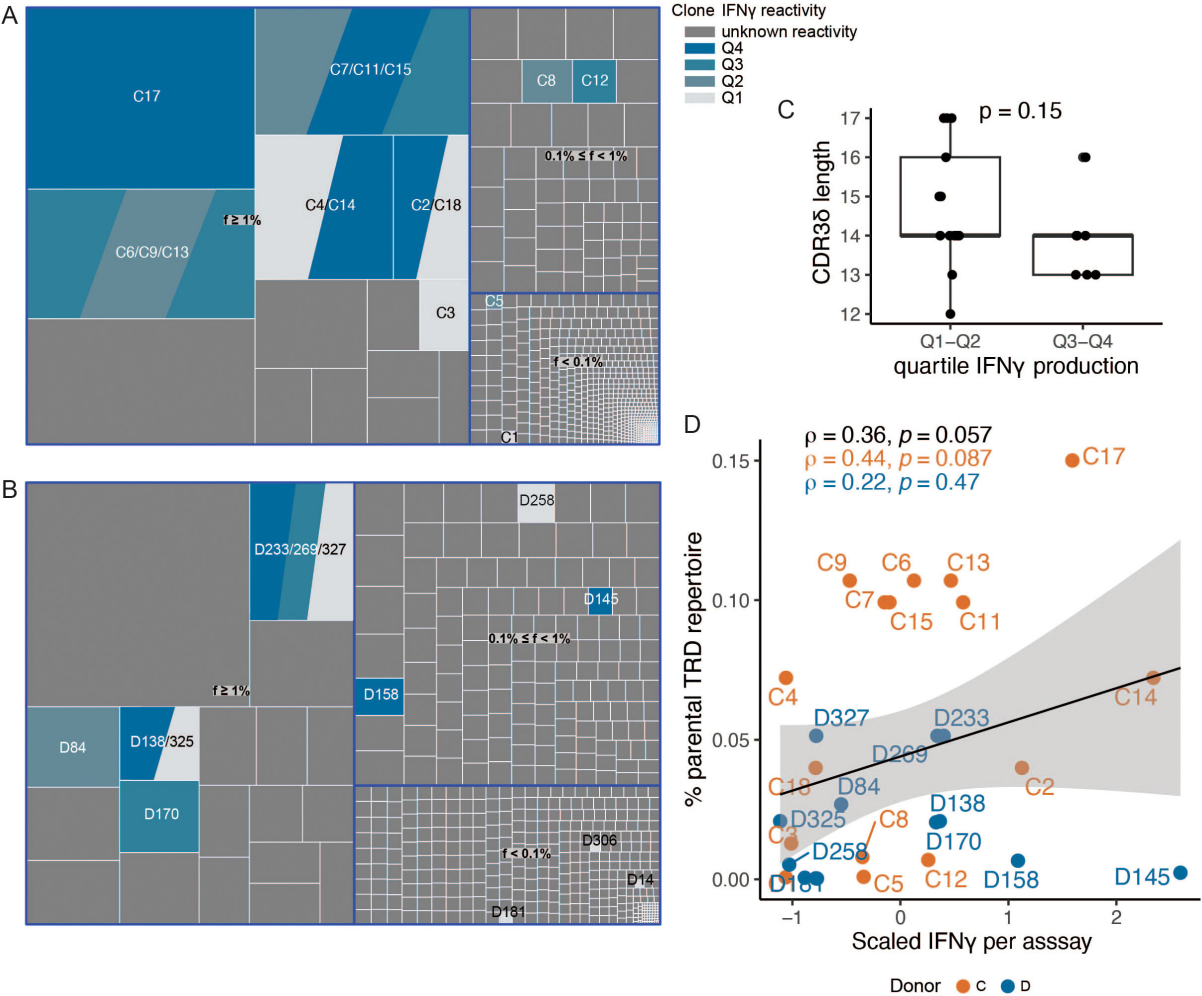
TCR repertoire analysis and estimation of *in vivo* expansion of the functionally profiled single cell clones. **(A, B)** Treemaps representing complete Vδ2 repertoires of the donors C **(A)** and D **(B)**, classified by clonotype frequency *f* (*f* ≥ 1%: hyperexpanded; 0.1% ≤ *f* < 1%: expanded; *f* < 0.1%: nonexpanded). Clones that were tested functionally are colored according to the quartile of IFN-γ production against HEK293FT cells. Clones sharing the same TCRδ chains are represented as occupying equal portions of the repertoire slot for the respective clonotype. **(C)** CDR3δ length (in amino acids) of clones grouped according to the quartile of IFN-γ production (target: HEK293FT cells). Included in the analysis are clones from donors C and D with no sharing of the TCRδ chain. *p*-value was calculated using the Wilcoxon rank-sum test. **(D)** IFN-γ secretion (target: HEK293FT cells) plotted against TRD clonotype frequency in the parental repertoire of the donors. Clones with shared TCRδ chains are plotted as multiple entries with the same clonotype frequency. Spearman correlation coefficients are reported.

Both the instruction of the naïve CD4 Th cells toward type 1 (IFN-γ) vs. type 2 (IL-4/IL-5/IL-13) effector programs, as well as IFN-γ secretion potential and polyfunctionality of the CD8 effector T cells, are in part shaped by the strength of the TCR signal (38, 39). To search for the origins of the differential activation and the resulting phenotypic and functional clusters of clones in the current dataset, we examined the CDR3δ features previously shown to contribute to TCRVγ9Vδ2 binding affinity and signaling strength, such as TRDJ1 region usage, presence of a hydrophobic amino acid at the position 5 (hAA5), and an optimal CDR3δ length (26, 40). To this end, we sequenced the TCRs of the individual clones (**Supplementary Table S2**). In order to deduce the clonotype frequencies of our functionally profiled single-cell clones in the parental repertoires, we additionally sequenced the bulk Vδ2 repertoires of the respective donors (**Figures 3A, B**).

Not surprisingly, several dominant TRD clonotypes were “sampled” multiple times during single-cell sorting (clones C2/C18, C4/C14, C6/9/13, C7/11/15, D138/325, and D233/269/327). While members of some of the resulting clone “families” showed near-identical functional profiles (such as C7/11/15 and C6/9), others (i.e. C4/14 and D233/327) belonged to either HIR or LIR group of clones, suggesting that a clonal population diverges *in vivo* or under the identical culture conditions *in vitro* into subclones of distinct phenotype and function. When focusing on the clones exclusively associated with HIR or LIR clusters, we detected no skewing in J1 region usage or hAA5, as the dataset was saturated with TRDJ1-rearranged sequences and rearrangements containing the amino acids L, V, or I (**Supplementary Figures S3A, B**). However, we noted a tendency toward shorter CDR3 length in the HIR group (**Figure 3C**).

In our earlier study, we were unable to conclusively demonstrate an association between the IFN-γ production potential of a clone and the frequency of the respective TRD clonotype in the individual’s TCR repertoire (26). Here, however, based on the pooled data from donors C and D, a subtle yet positive association emerged between the IFN-γ response and the clonotype frequency (HEK293FT + PAM stimulation condition, “clone families” are plotted as two or three individual clones with the same frequency, **Figure 3D**). Matching the transcriptional signature of the HIR cluster, which emphasized their highly proliferative nature, virtually all IFN-γ-producing clones showed some degree of clonal expansion (**Figures 3A, B, D**).

### Clone-intrinsic HIR and LIR signatures exhibit distinct degrees of phenotypic plasticity

**Figure 4:**
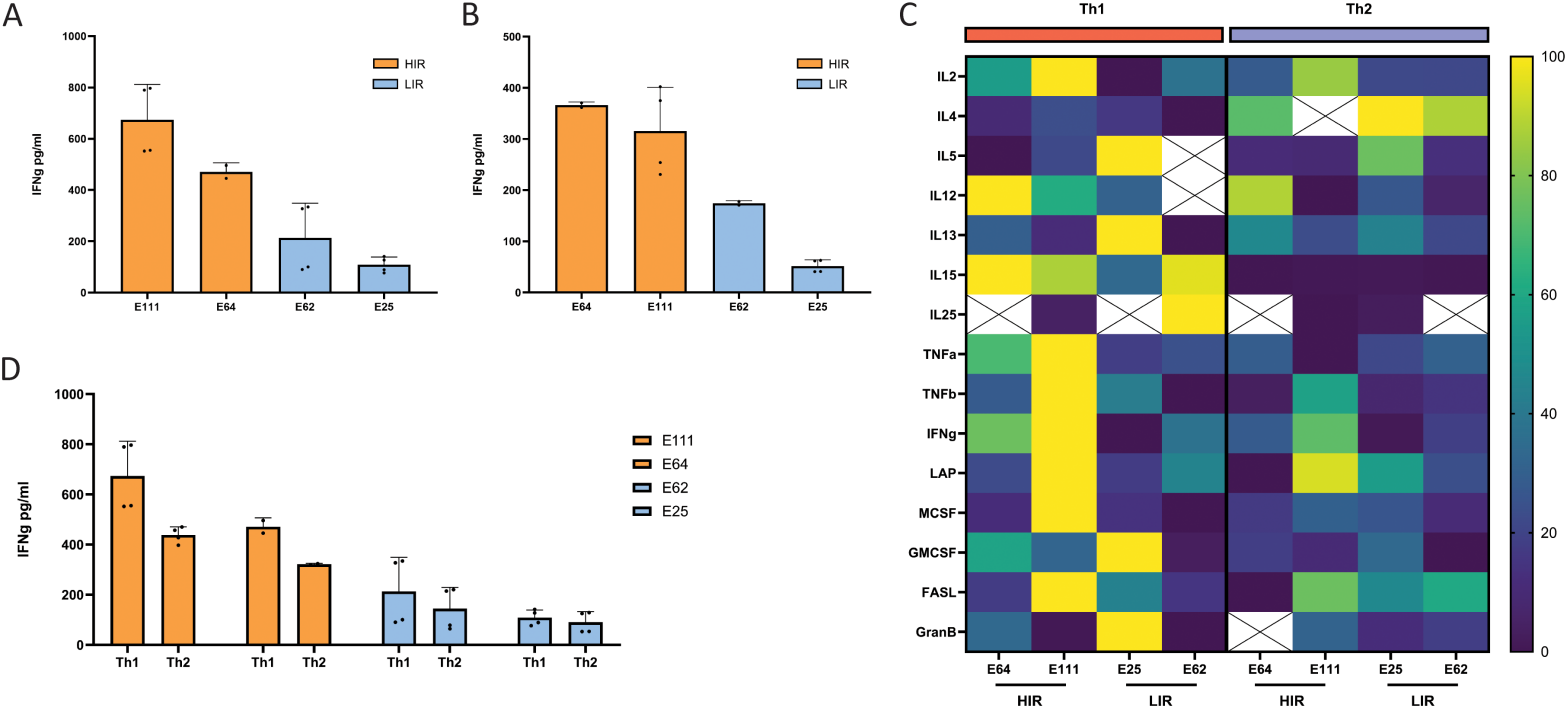
Phenotypic plasticity of HIR and LIR signatures. IFN-γ release concentration of expanded donor E Vγ9Vδ2 T-cell clones upon overnight coculture with HEK293FT **(A)** and Daudi **(B)** at an E:T ratio of 1:1 with 100 µM PAM, measured by ELISA. HIR/LIR signatures are determined according to the magnitude of IFN-γ release. **(C)** Heatmap of an array of cytokine concentrations secreted by donor E Vγ9Vδ2 T-cell clones cultured in both Th1- (red) and Th2-polarizing (blue) REP upon overnight coculture with HEK293FT at a 1:1 ratio with the addition of 100 µM PAM, normalized to each cytokine concentration. Blank cells represent measurements outside the range of the standard curve. **(D)** ELISA IFN-γ concentration of Vγ9Vδ2 T-cell clones challenged with HEK293FT overnight (E:T of 1:1) with 100 µM PAM after being cultured in Th1- (IL-2 and IL-15 added) or Th2-polarizing (IL-2, IL-4, and IL-13 added) REP conditions. Orange and blue represent HIR and LIR signatures, respectively.

To further emphasize interdonor consistency of the observed HIR and LIR signatures and to investigate phenotypic stability, we expanded additional Vγ9Vδ2 T-cell clones from an independent donor (donor E). Upon challenge with either HEK293FT (**Figure 4A**) or Daudi tumor cells (**Figure 4B**), a similar IFN-γ release gradient was observed, with a clear distinction between high- and low-releasing clones, mirroring the findings in donors C and D. As expected, production of the Th2-effector cytokines IL-5 and IL-13 was enriched in LIR clones, consistent with previous observations; however, in clones expanded from this donor, IL-4 production was more uniformly distributed across HIR and LIR clones. Conversely, higher production of TNF-α, IL-2, and IL-15 was observed in HIR clones, further validating the association between a Th1-effector cytokine profile and high IFN-γ release (**Figure 4C**). Differentially expressed genes at the transcriptional level that appeared promising as potential markers of HIR and LIR signatures, such as TIGIT, ICOS, and CCR3, did not show clear segregation at the protein level between signatures (**Supplementary Figures S4A–D**), implying a graded rather than binary expression pattern and reflecting heterogeneous positioning of individual clones along a continuous activation spectrum.

To assess the phenotypic plasticity of HIR and LIR clones in different environments, clones were further expanded under Th1- or Th2-polarizing culture conditions. The LIR phenotypic signature remained unaffected under Th1 conditions, as the low IFN-γ release profile was preserved. Under Th2-polarizing conditions, IFN-γ release by HIR clones was slightly reduced but remained substantially higher than that of LIR clones (**Figure 4D**). Moreover, the Th1 effector profile of HIR clones cultured under Th2 conditions appeared to skew, but not fully switch, toward a Th2-like effector state: TNF-α production was decreased, IL-2 release remained higher than in LIR clones, and IL-4 and IL-13 production were enhanced (**Figure 4C**). Together, these data indicate that both HIR and LIR signatures are largely clone-intrinsic, with LIR clones exhibiting a rigid Th2-like phenotype, whereas HIR clones display limited phenotypic plasticity within an overall Th1 effector profile.

### *In vivo* Vγ9Vδ2 T-cell heterogeneity reflects a transcriptional continuum between HIR and LIR states

**Figure 5:**
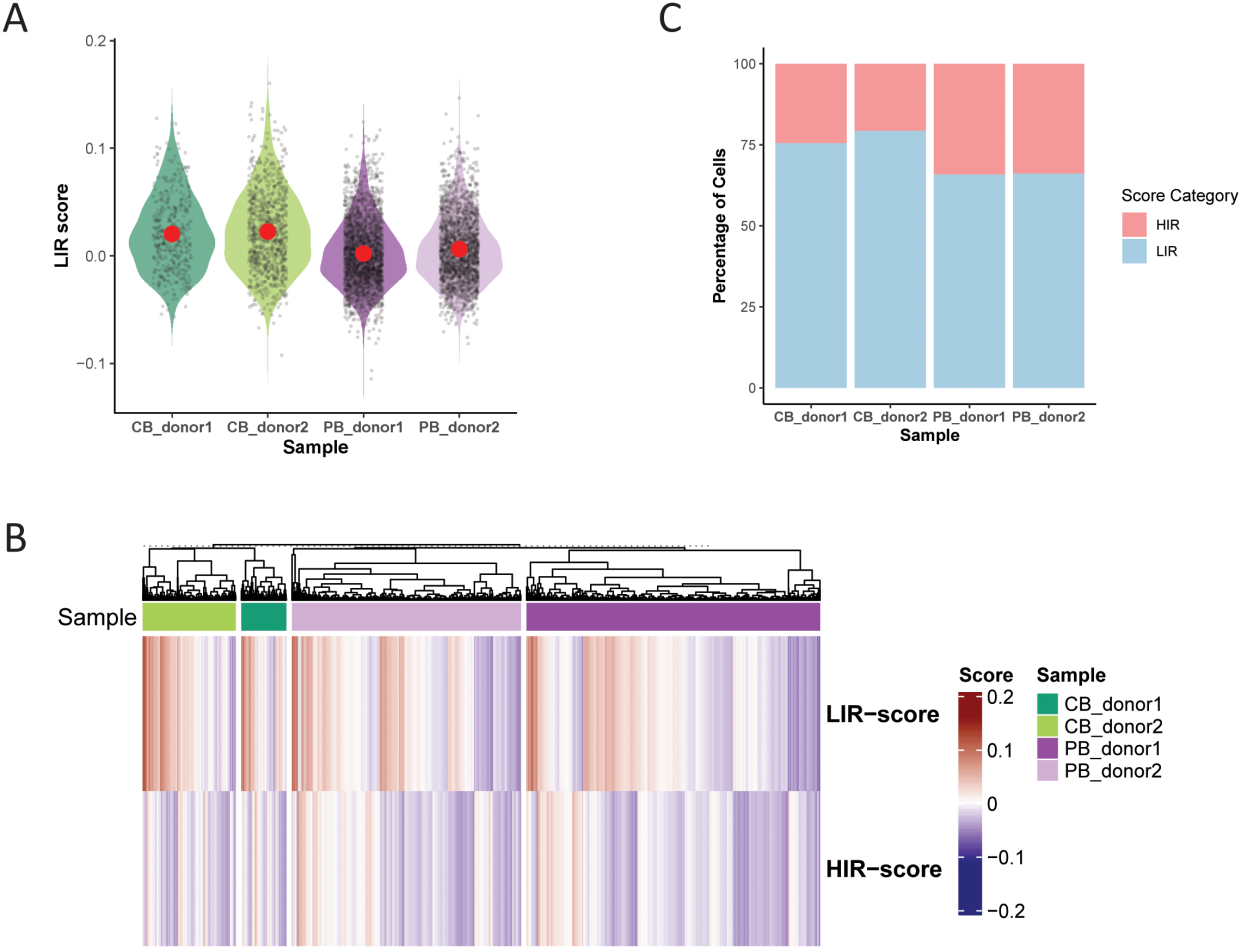
HIR and LIR transcriptional states form a pre-existing activation gradient in human Vγ9Vδ2 T cells. **(A)** Violin plots showing LIR module scores in Vγ9Vδ2 T cells from four individual donors (CB, cord blood; PB, peripheral blood). Each dot represents a single cell; red dots indicate the group mean. Cells were subsetted from GSE149356 based on TRGV9 and TRDV2 expression. **(B)** Heatmap of module scores (LIR, HIR) across individual cells grouped by donor origin. Samples are color-coded by donor type: cord blood (CB_donor1 and CB_donor2) and peripheral blood (PB_donor1 and PB_donor2). **(C)** Proportion of cells per sample classified as LIR- or HIR-dominant, based on module score comparison. Cells were classified in a mutually exclusive, threshold-free manner by comparing the relative magnitude of LIR and HIR module scores per cell.

To assess whether the *in vitro*-observed HIR and LIR signatures represent a culture-induced artifact or reflect preexisting *in vivo* states, we projected the HIR and LIR gene signatures onto a previously published single-cell transcriptomic dataset of nonexpanded Vγ9Vδ2 T cells (GSE149356 (17), subsetted based on TRGV9 and TRDV2 expression). This analysis revealed a pattern of enrichment of the two signatures both across and within donor samples. Consistent with a transcriptionally quiescent state, LIR scores were elevated in cord blood-derived cells (**Figure 5A**). Notably, both signatures displayed substantial cell-to-cell variability within individual samples (**Figure 5B**). To stratify cells more directly, each cell was classified as HIR- or LIR-dominant based on relative module scores. This approach revealed that LIR-dominant cells were significantly more abundant in cord blood samples, accounting for approximately 75% of cells in both cord blood_donor1 and cord blood_donor2, compared with ~ 66% and ~ 65% in PB_donor2 and PB_donor1, respectively (**Figure 5C**). Statistical analysis using Fisher’s exact test confirmed a significant association between sample origin and LIR dominance (*p* < 2.2 × 10^−16^), supporting the notion that cord blood-derived cells are transcriptionally biased toward a LIR-like state. Together, these findings support a model in which the HIR/LIR dichotomy observed following *in vitro* expansion reflects an underlying, cell-intrinsic transcriptional gradient already present *ex vivo*. The distribution of HIR and LIR states across all donors suggests that this gradient is neither discrete nor sample-restricted, but instead represents a continuum of preexisting transcriptional states that become stabilized upon culturing.

## Discussion

Mixed long-term responses observed in most hematologic malignancies treated with currently approved CAR T-cell products, as well as the poor amenability of solid tumors to targeting with ACT approaches, demand deeper insight into the heterogeneity of both the targets and the effectors (2, 5). Various aspects of carrier T-cell biology, such as stemness and the resulting proliferation capacity and longevity vs. effectorness and the exact effector flavor, have been shown to determine ACT outcomes. With Vγ9Vδ2 T cells in mind as a potential novel off-the-shelf ACT carrier, we set out to characterize the functional and transcriptomic features of Vγ9Vδ2 T-cell clones after rapid expansion *in vitro*. Here, we demonstrate partitioning of the *in vitro* REP-expanded Vγ9Vδ2 T-cell clones into HIR (“innate type 1”) and LIR (type 2-skewed) clusters, despite equal type 1 polarizing culture conditions (24). Importantly, rather than being mutually exclusive, the type 1 and type 2 effector programs were both present but antiparallel, akin to the effector fate gradients in CD4^+^ effectors (33), and were also observed in unexpanded PB mononuclear cells and cord blood, where LIR-like states predominated.

Probing the functionality and the transcriptome of expanded Vγ9Vδ2 T cells at the level of the single-cell clones revealed perfect correlations between secretion of multiple cytokines in response to tumor challenge and the storage pools of the respective cytokine mRNA at rest, in line with a broader view of the Vγ9Vδ2 T-cell population as innate and preprogrammed effectors (10). Cytokine responses to the cell lines of nonhematopoietic (HEK293FT) and hematopoietic (Daudi) origin were highly concordant, providing additional evidence that the clones are “intrinsically set” for a specific fate. A strong IFN-γ response was predictive of polyfunctionality among the Vγ9Vδ2 T-cell clones, both in terms of the number of effector molecules secreted upon challenge and the total cytokine mass. Moreover, polyfunctional HIR cells were characterized by a strong proliferative signature.

Reflecting on the origins of the observed transcriptional and functional diversity, two explanations are possible: (i) the cells are naturally “hardwired”, and the profiles obtained during *in vitro* culture reflect *in vivo* heterogeneity, or (ii) the profiles result from selection pressure in culture. By projecting HIR and LIR transcriptional signatures onto *ex vivo* single-cell transcriptomic data from nonexpanded Vγ9Vδ2 T cells, we demonstrate that these states are already represented *in vivo* as part of a continuous activation landscape. LIR-like states are enriched in cord blood and remain prevalent in adult PB, whereas HIR-like states are more prominent, but still heterogeneous, among circulating effector populations. The coexistence of both states within individual donors, together with pronounced cell-to-cell variability, argues against a discrete lineage model and instead supports a gradient of preexisting activation states that are stabilized and functionally amplified during *in vitro* expansion.

Recent (sc)RNA seq studies of the γδ T cells have highlighted substantial transcriptional and phenotypic heterogeneity within the γδ T-cell compartment, where most adult PB Vγ9Vδ2 T cells can be classified as CTL/type 1 immature or mature effectors (11, 17–19, 41, 42). Type 2-like transcriptional programs have been documented primarily in neonates, accounting for approximately 8%–12% of γδ T cells in cord blood, while being virtually absent from adults (1%–2% of PB γδ T cells) (17), consistent with the identification of type 2-like transcriptional programming in maturing γδ thymocytes during fetal development and its absence after birth (41). In our analysis, however, LIR-like transcriptional states were frequent in nonexpanded PBMCs and, although reduced from cord blood to adult PB, remained the dominant population. This apparent discrepancy in the reported frequency of type 2-like states when compared to other studies (11, 17–19, 41, 42) likely reflects differences in how transcriptional states were defined and, importantly, the lack of direct functional anchoring in prior analyses. Indeed, previous studies generally failed to demonstrate IL-4 or IL-5 expression at the protein level in putative type 2-programmed γδ T-cells *ex vivo*, leaving the question of true functional commitment unresolved (19). By directly linking transcriptional profiles to cytokine production at the clonal level, our data suggest that the functional relevance of type 2-skewed states in adult Vγ9Vδ2 T cells may have been underestimated. Although we could not confirm differential expression of all proposed surface markers, such as TIGIT, ICOS, and CCR3, this likely reflects interclonal variability combined with the limited number of expanded clones analyzed, as well as the existence of a continuum of clonal phenotypes that are stabilized and amplified from preexisting transcriptional biases into overt functional states.

The Vγ9Vδ2TCR is a recombined receptor with a unique antigen specificity for a butyrophilin complex assembled by intracellular phosphoantigens derived from the mevalonate pathway (12). The Vγ9Vδ2TCR repertoire, although extremely diverse, has been deemed “semi-invariant” due to germline-encoded features of the Vγ9 chain and conserved CDR3γ structures (43, 44). In contrast, features of the more diverse CDR3δ, such as hydrophobic AA at the fifth position and CDR3δ length, have been shown to influence target binding (26, 40), signaling strength (45, 46), and effector differentiation (41). Importantly, previous work has demonstrated that these TCR features primarily affect activation magnitude and clonal expansion rather than irreversibly determining effector phenotype (26, 47). Seeking to disentangle intrinsic versus activation-driven contributions to the HIR/LIR dichotomy, we examined TCR features as clone-defining elements. No clear enrichment of hydrophobic amino acids at position five was observed in the HIR cluster, although we noted a trend toward shorter CDR3δ lengths. Notably, several isolated HIR and LIR clones shared identical TCR sequences. This observation is consistent with recent reports showing that phenotypic states do not strictly segregate by clonotype (17). Rather, individual clonotypes appear biased, but not fixed, toward particular activation and effector states, with TCR signaling strength acting as a modulatory trigger rather than a deterministic factor (17, 41). Accordingly, while shorter CDR3δ length in HIR clones may contribute to enhanced signaling and clonal expansion, our data support a model in which TCR affinity modulates position along a continuous activation landscape rather than dictating discrete HIR or LIR fate.

Importantly, recognition that HIR- and LIR-like states exist *in vivo* and *ex vivo* has direct implications for γδ T-cell engineering and for strategies aiming to amplify Vγ9Vδ2 T-cell function *in vivo* or *ex vivo* (8). Our data indicate that the majority of circulating Vγ9Vδ2 T cells reside in an LIR-like state *in vivo* and retain this state upon entry into *ex vivo* culture. Notably, antibody-mediated stimulation *in vivo* or *ex vivo* is most likely insufficient to override this endogenous effector programming, as evidenced by the persistence of LIR-like functional profiles under activating culture conditions. This challenges the assumption that strong pharmacologic or antibody-driven activation alone can reset the effector state. Similarly, introduction of a potent synthetic receptor, such as a CAR, is likely layered onto this preexisting activation landscape rather than replacing it, so most CAR-engineered γδ T-cell products may inherit LIR-like transcriptional and functional features unless active measures are taken to redirect them. Together, these observations may in part contribute to the variable potency and durability reported in early γδ CAR T-cell studies (8) and highlight baseline effector heterogeneity as an underappreciated determinant of product performance. This limitation may be overcome by alternative strategies, such as using αβ T cells as carriers for defined γδ TCRs, which display a more activated transcriptional and functional phenotype (48) and can be further enhanced through appropriate costimulatory signaling (49, 50).

In summary, while our study has an exploratory character and a limited sample size, we provide evidence for pronounced transcriptional and functional heterogeneity within the Vγ9Vδ2 T-cell compartment that is already present *in vivo* and is retained and amplified upon *ex vivo* expansion. By demonstrating that HIR- and LIR-like states form a preexisting continuum, we establish a unifying framework in which activation history, rather than discrete lineage commitment, governs γδ T-cell effector diversity. Although the subdivision into HIR and LIR clusters represents a simplification of the full diversity revealed by high-resolution single-cell analyses (17, 19, 41), it offers a practical and biologically grounded framework for studying γδ T-cell effector heterogeneity. Critically, recognizing that most Vγ9Vδ2 T cells enter manufacturing pipelines in a LIR-like state provides a strong rationale for deliberately steering activation, expansion, and engineering strategies toward HIR-associated phenotypes to maximize therapeutic efficacy in ACT applications.

## Data Availability

Raw FASTQ files from the RNA-seq dataset have been deposited in the Sequence Read Archive (SRA) under BioProject ID PRJNA1418759.
