# Supplementary material for: Pre-existing activation states shape functional heterogeneity of human Vγ9Vδ2 T cells
Source: Front Immunol. 2026 Feb 16;17:1696469. doi: 10.3389/fimmu.2026.1696469 (PMC12950705; doi:10.3389/fimmu.2026.1696469)
Supplement: Supplementary file 1 [file DataSheet1.pdf]

Supplementary Material for
Differential activation shapes the functional profile of in vitro Vγ9Vδ2T cell effectors.

Anna Vyborova, Laia Gasull-Celades, Peter Brazda, Alberto Miranda Bedate, Froso Karaiskaki, Jasper Sanders, Anke Janssen, Trudy Straetemans, Dennis Beringer, Zsolt Sebestyen, Jürgen Kuball

Corresponding author: Jürgen Kuball
Email: J.H.E.Kuball@umcutrecht.nl

This file includes:

Figures S1 to S4
Tables S1 to S2
References for supplementary reference citations

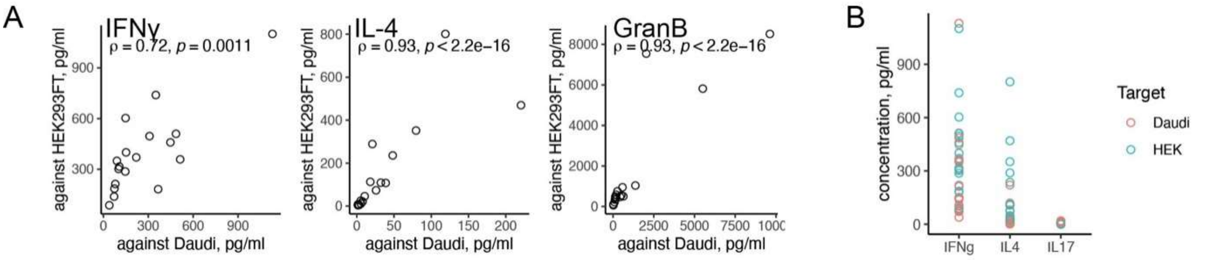

Supplementary Figure 1

A. Release of IFNγ, IL-4 and Granzyme B in response to Daudi (hematologic) and HEK293FT (embryonic kidney) cells (Donor C). **B.** Absolute concentrations of IFNγ, IL-4, and IL-17 in the supernatants from both targets measured by Luminex assay (Donor C).

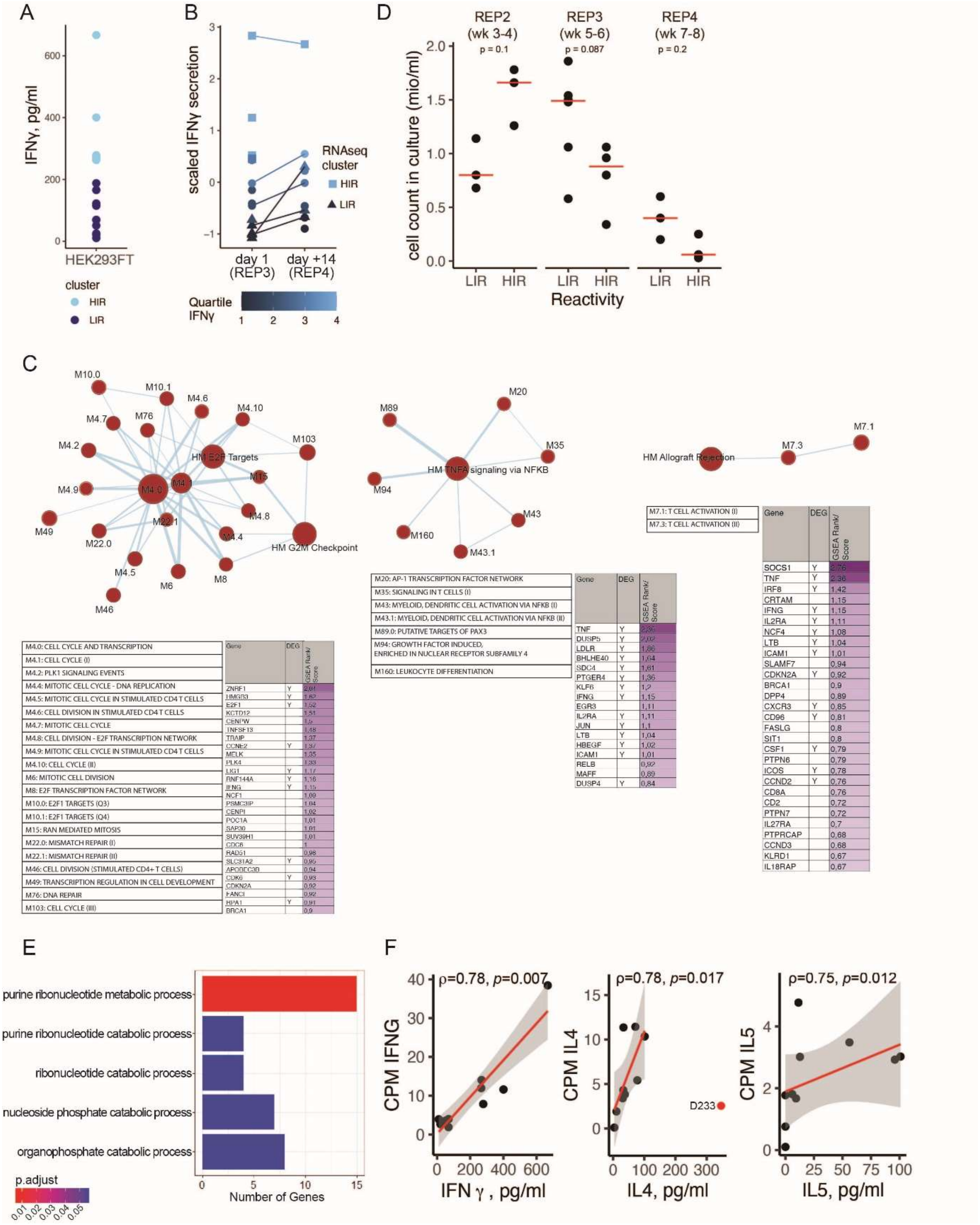

M4.0: CELL CYCLE AND TRANSCRIPTION
M4.1: CELL CYCLE (I)
M4.2: PLK1 SIGNALING EVENTS
M4.4: MITOTIC CELL CYCLE - DNA REPLICATION
M4.5: MITOTIC CELL CYCLE IN STIMULATED CD4 T CELLS
M4.6: CELL DIVISION IN STIMULATED CD4 T CELLS
M4.7: MITOTIC CELL CYCLE
M4.8: CELL DIVISION - E2F TRANSCRIPTION NETWORK
M4.9: MITOTIC CELL CYCLE IN STIMULATED CD4 T CELLS
M4.10: CELL CYCLE (II)
M6: MITOTIC CELL DIVISION
M8: E2F TRANSCRIPTION FACTOR NETWORK
M10.0: E2F1 TARGETS (Q3)
M10.1: E2F1 TARGETS (Q4)
M15: RAN MEDIATED MITOSIS
M22.0: MISMATCH REPAIR (I)
M22.1: MISMATCH REPAIR (II)
M46: CELL DIVISION (STIMULATED CD4+ T CELLS)
M49: TRANSCRIPTION REGULATION IN CELL DEVELOPMENT
M76: DNA REPAIR
M103: CELL CYCLE (III)

Gene	DEG	GSEA Rank/ Score
ZNRF1	Y	2,04
HMGB3	Y	1,62
E2F1	Y	1,52
KCTD12		1,51
CENPW		1,5
TNFSF13		1,48
TRAIP		1,37
CCNE2	Y	1,37
MELK		1,35
PLK4		1,33
LIG1	Y	1,17
RNF144A	Y	1,16
IFNG	Y	1,15
NCF1		1,09
PSMC3IP		1,04
CENPI		1,02
POC1A		1,01
SAP30		1,01
SUV39H1		1,01
CDC6		1
RAD51		0,98
SLC31A2	Y	0,95
APOBEC3B		0,94
CDK6	Y	0,93
CDKN2A		0,92
FANCI		0,92
RPA1	Y	0,91
BRCA1		0,9

M20: AP-1 TRANSCRIPTION FACTOR NETWORK
M35: SIGNALING IN T CELLS (I)
M43: MYELOID, DENDRITIC CELL ACTIVATION VIA NFKB (I)
M43.1: MYELOID, DENDRITIC CELL ACTIVATION VIA NFKB (II)
M89.0: PUTATIVE TARGETS OF PAX3
M94: GROWTH FACTOR INDUCED, ENRICHED IN NUCLEAR RECEPTOR SUBFAMILY 4
M160: LEUKOCYTE DIFFERENTIATION

Gene	DEG	GSEA Rank/ Score
TNF	Y	2,36
DUSP5	Y	2,02
LDLR	Y	1,86
BHLHE40	Y	1,64
SDC4	Y	1,61
PTGER4	Y	1,36
KLF6	Y	1,2
IFNG	Y	1,15
EGR3		1,11
IL2RA	Y	1,11
JUN	Y	1,1
LTB	Y	1,04
HBEGF	Y	1,02
ICAM1	Y	1,01
RELB		0,92
MAFF		0,89
DUSP4	Y	0,84

M7.1: T CELL ACTIVATION (I)
M7.3: T CELL ACTIVATION (II)

Gene	DEG	GSEA Rank/ Score
SOCS1	Y	2,76
TNF	Y	2,36
IRF8	Y	1,42
CRTAM		1,15
IFNG	Y	1,15
IL2RA	Y	1,11
NCF4	Y	1,08
LTB	Y	1,04
ICAM1	Y	1,01
SLAMF7		0,94
CDKN2A	Y	0,92
BRCA1		0,9
DPP4		0,89
CXCR3	Y	0,85
CD96	Y	0,81
FASLG		0,8
SIT1		0,8
CSF1	Y	0,79
PTPN6		0,79
ICOS	Y	0,78
CCND2	Y	0,76
CD8A		0,76
CD2		0,72
PTPN7		0,72
IL27RA		0,7
PTPRCAP		0,68
CCND3		0,68
KLRD1		0,67
IL18RAP		0,67

G

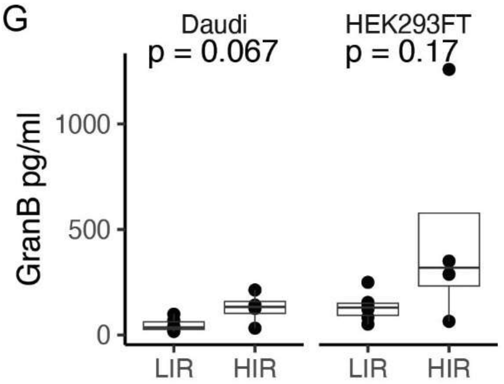

Supplementary Figure 2

A. HEK293FT-induced IFNγ production by the clones isolated from donor D. K-means clustering algorithm with k = 2 was used to divide the pool of clones into HIR and LIR clusters. **B.** HEK293FT-induced IFNγ production by the clones isolated from donor D; clones were tested functionally after two consecutive REP rounds. Colors represent magnitude of IFNγ release, lines connect the clones which were tested twice. Clones which were selected for the RNAseq experiment are specified using shapes (HIR = square, LIR = triangle). **C.** Enrichment map of the MSigDB hallmark pathways and BTMs (selected by p<0.1 and FDRq<0.25), as well as the key genes in each of the nodes. Other pathways appearing as single nodes without connections were: HALLMARK_INTERFERON_GAMMA_RESPONSE, HALLMARK_APOPTOSIS, HALLMARK_P53_PATHWAY, REGULATION OF ANTIGEN PRESENTATION AND IMMUNE RESPONSE (M5.0), MHC-TLR7-TLR8 CLUSTER (M146), PLASMA CELLS, IMMUNOGLOBULINS (M156.1), HALLMARK_HEDGEHOG_SIGNALING, ENRICHED IN DNA INTERACTING PROTEINS (M182), ANTIGEN PRESENTATION (LIPIDS AND PROTEINS) (M28), TBA (M136). **D.** T cell expansion during culture approximated by reached peak cell density. Successive REPs are shown, cell count in the culture flasks is plotted. **E.** Gene Ontology (GO) Biological Processes annotation of the DEGs in LIR cells. **F.** HEK293FT-induced IFNγ, IL4 and IL5 secretion (pg/ml) vs mRNA storage (CPM) in the clones from donor D. **G.** In vitro Granzyme B secretion by the HIR/LIR clones upon overnight challenge with the tumor cell lines.

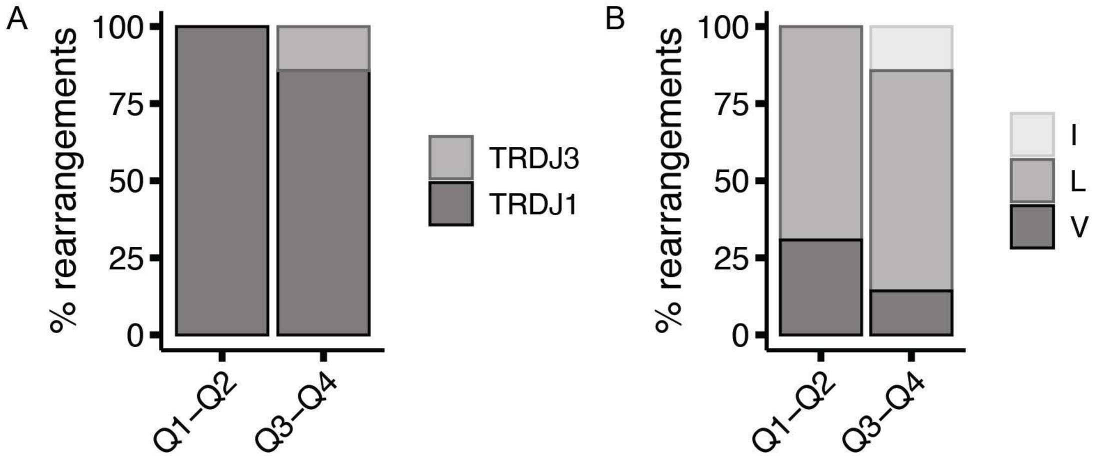

Supplementary Figure 3
A. CDR3δ J1 region usage, and **B.** Percentage rearrangements featuring hydrophobic amino acids L,V,I at position 5 of the CDR3δ. Clones are grouped according to the quartile of IFNγ production (donors C and D, clones with unique CDR3δ sequences are included in the analysis; target: HEK293FT cells).

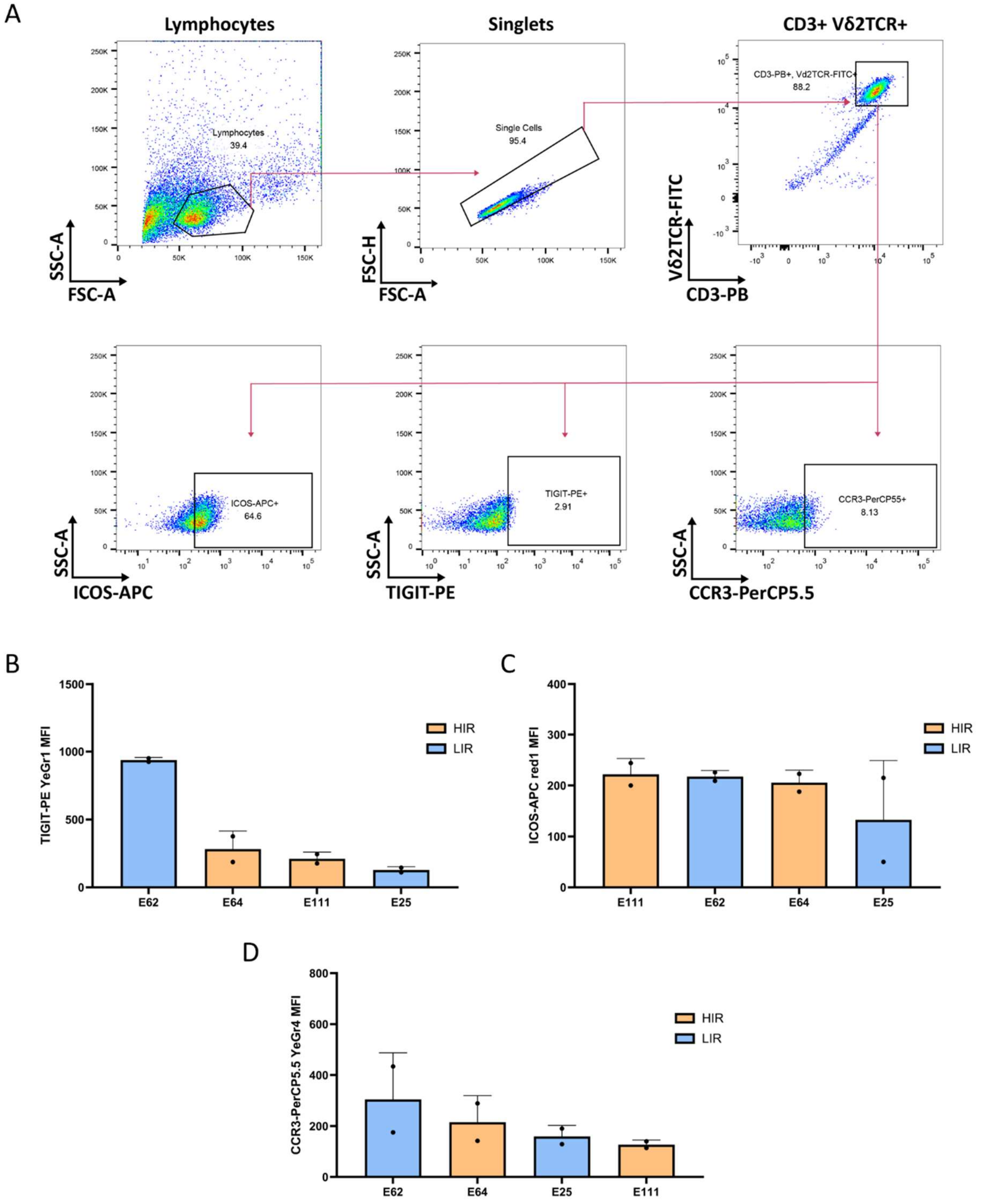

Supplementary Figure 4

A. Flow cytometry gating strategy for marker expression analysis of Vγ9Vδ2 T cell clones of donor E after Th1-polarizing REP expansion. Protein expression shown as mean MFI of (**B**) TIGIT-PE (Clone MBSA43, #12-9500-42), (**C**) ICOS-APC (Clone ISA3, #17-9948-42), (**D**) CCR3-PerCP5.5 (Clone 5E8, #2153590). HIR/LIR signature labels were determined previously according to IFNγ release magnitude.

Supplementary Table 1. DEG analysis results.

ensembl	baseMean	log2FoldChange	lfcSE	stat	pvalue	padj	entrez	hgnc_symbol
ENSG00000205403	9.924.557.681	-5.456.227.514	1.306.306.527	-4.176.835.528	2,96E+00	0.001562464	3426	CFI
ENSG00000181847	5.502.384.348	-4.945.013.573	1.102.073.804	-4.487.007.634	7,22E-01	0.000529533	201633	TIGIT
ENSG00000163554	1.351.787.177	-4.905.083.622	1.244.850.535	-3.940.299.245	8,14E-02	0.003411647	6708	SPTA1
ENSG00000064300	2.680.383.221	-4.741.860.357	0.921243309	-5.147.239.944	2,64E-02	3,82E+00	4804	NGFR
ENSG00000204528	1.108.249.188	-4.635.960.097	1.282.826.305	-361.386.423	0.000301667	0.009057201	NA	PSORS1C3
ENSG00000203747	1.758.081.275	-4.336.833.384	109.553.691	-3.958.637.397	7,54E+00	0.003230997	2214	FCGR3A
ENSG00000244682	1.643.872.977	-3.853.381.385	0.909936569	-4.234.780.221	2,29E+00	0.001288238	NA	FCGR2C
ENSG00000133142	4.034.005.934	-3.800.125.736	0.721460265	-5.267.269.617	1,38E-02	2,20E+00	79921	TCEAL4
ENSG00000246731	5.628.234.157	-3.311.887.227	0.627711686	-5.276.128.036	1,32E-02	2,12E+00	85001	
ENSG00000172465	4.911.594.165	-3.097.879.096	0.50723044	-6.107.439.248	1,01E-04	3,36E-02	9338	TCEAL1
ENSG00000171408	3.196.520.426	-3.043.166.482	0.594744157	-5.116.765.667	3,11E-02	4,36E+00	27115	PDE7B
ENSG00000102409	1.367.355.423	-3.002.850.046	0.578954425	-5.186.677.771	2,14E-02	3,22E+00	56271	BEX4
ENSG00000165511	1.351.754.614	-2.719.593.177	0.73374727	-3.706.444.016	0.00021019	0.006860083	220979	C10orf25
ENSG00000130413	3.668.379.875	-2.637.946.142	0.421102871	-6.264.374.638	3,74E-05	1,61E-02	65975	STK33
ENSG00000164112	9.617.808.693	-2.553.673.326	0.69652685	-3.666.295.599	0.000246089	0.00780478	132332	TMEM155
ENSG00000271270	3.687.102.064	-2.190.060.589	0.54102453	-40.479.876	5,17E+00	0.002456585	1,01E+08	TMCC1-AS1
ENSG00000211666	3.754.169.415	-2.173.899.891	0.339519919	-640.286.407	1,52E-05	7,42E-03	NA	IGLV2-14
ENSG00000241490	1.733.614.895	-2.163.371.201	0.321043674	-6.738.557.328	1,60E-06	1,23E-03	NA	
ENSG00000253590	3.163.454.385	-2.149.340.095	0.356188339	-6.034.279.784	1,60E-04	5,06E-02	NA	IGLV3-13
ENSG00000146858	3.763.822.776	-2.138.455.431	0.201992418	-1.058.681.042	3,43E-21	5,01E-17	92092	ZC3HAV1L
ENSG00000206077	1.398.543.996	-2.026.203.456	0.537697387	-3.768.297.012	0.000164365	0.005722964	653082	ZDHHC11B
ENSG00000211667	2.495.846.972	-1.959.472.926	0.36257073	-5.404.388.064	6,50E-05	1,13E+00	NA	IGLV3-12
ENSG00000007350	2.775.411.734	-1.959.288.683	0.33896719	-5.780.172.064	7,46E-04	1,85E-01	8277	TKTL1
ENSG00000154269	5.132.944.032	-1.921.440.657	0.407750759	-4.712.292.049	2,45E-01	0.000233617	5169	ENPP3
ENSG00000131459	597.239.694	-1.871.948.196	0.473863815	-3.950.392.782	7,80E-01	0.003295649	9945	GFPT2
ENSG00000095539	2.104.268.724	-1.852.814.784	0.51045585	-3.629.725.827	0.000283722	0.008635417	57715	SEMA4G
ENSG00000179111	5.464.960.377	-182.991.716	0.403225746	-4.538.195.237	5,67E-01	0.000442645	84667	HES7

ENSG00000231028	6.526.509.143	-177.330.064	0.318557303	-5.566.661.396	2,60E-03	5,49E-01	1E+08	LINC00271
ENSG00000123191	2.155.580.133	-1.720.374.906	0.412680101	-4.168.785.708	3,06E+00	0.001601269	540	ATP7B
ENSG00000141540	1.065.148.827	-1.717.911.134	0.361094677	-475.750.889	1,96E-01	0.000191906	94015	TTYH2
ENSG00000168811	4.100.479.077	-1.683.836.771	0.404587204	-4.161.863.631	3,16E+00	0.001633041	3592	IL12A
ENSG00000163568	1.784.174.962	-1.674.987.141	0.288361781	-5.808.630.855	6,30E-04	1,61E-02	9447	AIM2
ENSG00000115657	1.888.270.373	-1.618.942.451	0.382391732	-4.233.727.656	2,30E+00	0.001288238	10058	ABCB6
ENSG00000130595	1.343.276.364	-1.609.651.944	0.447797912	-3.594.594.571	0.000324897	0.009425673	7140	TNNT3
ENSG00000123384	3.788.971.975	-1.597.820.251	0.42028154	-3.801.785.468	0.000143657	0.00514995	4035	LRP1
ENSG00000231830	1.310.824.207	-1.543.676.977	0.427345974	-3.612.241.771	0.000303561	0.009057201	NA	
ENSG00000165113	8.668.815.064	-1.540.509.086	0.176297845	-8.738.105.012	2,37E-13	5,76E-10	80318	GKAP1
ENSG00000254911	73.158.472	-15.151.026	0.385442095	-3.930.817.674	8,47E+00	0.003538876	1E+08	SCARNA9
ENSG00000267566	1.523.825.694	-1.508.764.679	0.354710035	-4.253.515.628	2,10E-01	0.001213483	NA	NA
ENSG00000266956	2.895.527.324	-1.483.321.007	0.296322907	-5.005.758.824	5,56E-02	6,49E+00	NA	NA
ENSG00000267454	1.188.352.832	-1.437.793.191	0.399971226	-3.594.741.568	0.000324714	0.009425673	386758	ZNF582-AS1
ENSG00000163975	4.819.965.623	-1.354.903.606	0.271977429	-498.167.665	6,30E-02	7,13E-01	4241	MELTF
ENSG00000182749	489.143.872	-1.346.568.216	0.329673464	-4.084.551.424	4,42E-01	0.002154783	164091	PAQR7
ENSG00000080854	7.038.223.676	-134.399.146	0.286030513	-4.698.769.541	2,62E-01	0.000241673	22997	IGSF9B
ENSG00000013016	1.968.319.873	-1.327.200.475	0.338564577	-3.920.080.728	8,85E+00	0.003648444	30845	EHD3
ENSG00000215424	1.260.903.875	-1.311.303.696	0.299329147	-4.380.808.588	1,18E-01	0.000787671	114044	MCM3AP-AS1
ENSG00000119729	3.942.551.448	-1.287.118.728	0.343441882	-3.747.704.623	0.00017846	0.006070478	23433	RHOQ
ENSG00000124570	9.001.532.946	-1.260.553.086	0.254372875	-4.955.532.649	7,21E-02	7,97E+00	5269	SERPINB6
ENSG00000049192	1.497.142.028	-1.242.131.329	0.273993262	-4.533.437.499	5,80E-01	0.000447111	11174	ADAMTS6
ENSG00000204991	1.522.616.151	-1.228.950.733	0.331473661	-370.753.661	0.000209285	0.006845877	84501	SPIRE2
ENSG00000135541	2.002.248.582	-1.214.077.098	0.320514807	-3.787.897.065	0.000151928	0.005379792	54806	AHI1
ENSG00000108622	2.724.485.184	-1.205.467.105	0.322783074	-373.460.445	0.00018801	0.006334606	3384	ICAM2
ENSG00000137747	1.734.309.837	-11.556.957	0.184932904	-6.249.270.269	4,12E-05	1,67E-02	84000	TMPRSS13
ENSG00000227053	3.106.958.745	-1.112.227.461	0.249156561	-4.463.970.198	8,05E-01	0.000576585	1,03E+08	
ENSG00000271614	7.901.315.777	-1.104.968.596	0.247555514	-446.351.841	8,06E-01	0.000576585	NA	ATP2B1-AS1
ENSG00000256073	4.475.306.562	-1.073.304.429	0.29166029	-367.998.135	0.000233251	0.007511918	84996	URB1-AS1

ENSG00000272941	4.099.240.354	-1.041.730.714	0.253911661	-410.272.891	4,08E+00	0.002019244	NA	
ENSG00000242258	7.266.278.046	-1.032.440.441	0.219147273	-4.711.171.749	2,46E-01	0.000233617	285972	LINC00996
ENSG00000116667	1.808.261.522	-0.98021684	0.267270575	-36.675.075	0.000244926	0.007784817	81563	C1orf21
ENSG00000186854	3.719.578.927	-0.978539052	0.260765132	-3.752.568.628	0.000175032	0.00600833	1,05E+08	TRABD2A
ENSG00000185989	5.736.006.444	-0.954391553	0.263668393	-3.619.666.133	0.000294983	0.008909964	22821	RASA3
ENSG00000083290	3.278.058.628	-0.940225594	0.22191515	-423.686.979	2,27E+00	0.001281669	9706	ULK2
ENSG00000234231	1.082.872.039	-0.904655331	0.232554843	-3.890.073.063	0.000100214	0.003994598	NA	
ENSG00000135362	2.816.454.475	-0.896853613	0.187364565	-4.786.676.783	1,70E-01	0.000171791	79899	PRR5L
ENSG00000068024	7.232.384.382	-0.895356732	0.234229832	-382.255.635	0.000132075	0.004853517	9759	HDAC4
ENSG00000177383	1.568.162.305	-0.887484268	0.241047021	-3.681.788.988	0.000231603	0.007475348	64110	MAGEF1
ENSG00000068976	6.451.951.638	-0.873094565	0.203067659	-4.299.525.446	1,71E+00	0.001040465	5837	PYGM
ENSG00000115306	3.821.364.527	-0.864899102	0.18390031	-4.703.086.695	2,56E-01	0.000240945	6711	SPTBN1
ENSG00000273008	3.998.499.059	-0.864045923	0.216502418	-3.990.929.666	6,58E+00	0.002936306	NA	
ENSG00000146540	660.291.955	-0.851757473	0.231660915	-3.676.742.238	0.000236232	0.007574465	84310	C7orf50
ENSG00000165322	5.469.076.528	-0.851173233	0.150225396	-5.665.974.319	1,46E-03	3,18E-01	94134	ARHGAP12
ENSG00000237172	1.953.102.423	-0.849969904	0.20808515	-4.084.721.594	4,41E+00	0.002154783	84752	B3GNT9
ENSG00000183386	229.664.246	-0.84081665	0.189775686	-4.430.581.525	9,40E-01	0.000649793	2275	FHL3
ENSG00000114270	3.940.592.014	-0.8403134	0.202958049	-4.140.330.496	3,47E+00	0.001750709	1294	COL7A1
ENSG00000224616	4.805.122.768	-0.839969618	0.230036877	-365.145.637	0.000260757	0.008163497	NA	RTCA-AS1
ENSG00000163421	5.376.380.883	-0.825410411	0.191295886	-4.314.836.177	1,60E+00	0.000987362	60675	PROK2
ENSG00000248367	8.512.850.535	-0.811041737	0.183868834	-4.410.979.924	1,03E+00	0.000698263	NA	
ENSG00000249454	4.292.116.272	-0.804799797	0.205303219	-3.920.054.438	8,85E-01	0.003648444	NA	GZMAP1
ENSG00000185513	4.395.357.366	-0.799178749	0.171083735	-4.671.272.525	2,99E-01	0.00026171	26013	L3MBTL1
ENSG00000272906	3.601.034.625	-0.782940785	0.218629256	-3.581.134.558	0.000342105	0.009751791	NA	
ENSG00000152556	2.678.265.227	-0.759774595	0.20728853	-3.665.299.732	0.000247049	0.007818219	5213	PFKM
ENSG00000144451	1.118.432.145	-0.751193727	0.205976031	-3.646.995.826	0.000265324	0.008218291	79582	SPAG16
ENSG00000146859	1.356.499.128	-0.729832394	0.181206502	-4.027.628.074	5,63E+00	0.002617767	55281	TMEM140
ENSG00000162894	2.792.888.222	-0.729398718	0.182439203	-3.998.037.188	6,39E+00	0.002867072	9214	FCMR
ENSG00000164649	7.654.414.403	-0.721150966	0.185627311	-3.884.939.996	0.000102355	0.00405777	55536	CDCA7L

ENSG00000188825	1.781.862.143	-0.701099904	0.181793056	-385.658.243	0.000114983	0.004402866	NA	LINC00910
ENSG00000133466	1.358.995.155	-0.68748934	0.138168883	-4.975.717.583	6,50E-02	7,30E+00	114904	C1QTNF6
ENSG00000160746	3.595.152.499	-0.684201238	0.165451686	-4.135.353.668	3,54E+00	0.001776792	55129	ANO10
ENSG00000133624	1.824.591.148	-0.678288685	0.132814026	-5.107.056.131	3,27E-02	4,46E+00	79970	ZNF767P
ENSG00000154930	3.889.991.275	-0.674894379	0.074024166	-9.117.216.874	7,71E-15	2,81E-11	84532	ACSS1
ENSG00000140876	9.273.025.636	-0.669188578	0.177970473	-3.760.110.136	0.000169839	0.005857624	283927	NUDT7
ENSG00000260563	3.150.992.201	-0.667546552	0.138896619	-4.806.067.696	1,54E-01	0.000157039	NA	
ENSG00000107485	1.638.992.168	-0.666050967	0.131018774	-5.083.629.976	3,70E-02	4,82E+00	2625	GATA3
ENSG00000198933	1.447.755.818	-0.66467558	0.143434842	-4.633.989.685	3,59E-01	0.000304236	9755	TBKBP1
ENSG00000206344	1.921.208.268	-0.664007576	0.176368251	-3.764.892.901	0.00016662	0.005760252	253018	HCG27
ENSG00000232533	1.246.158.452	-0.663092124	0.145543171	-4.555.982.375	5,21E-01	0.000411184	NA	
ENSG00000231999	1.030.753.417	-0.658673949	0.167649691	-3.928.870.641	8,53E+00	0.003557454	400761	LRRC8C-DT
ENSG00000171115	2.295.000.789	-0.649293179	0.138482572	-4.688.627.397	2,75E-01	0.000247692	155038	GIMAP8
ENSG00000172794	4.754.252.668	-0.633585701	0.146553184	-4.323.247.589	1,54E+00	0.000971015	326624	RAB37
ENSG00000004478	9.709.486.822	-0.623230496	0.170864045	-3.647.522.765	0.000264781	0.008218291	2288	FKBP4
ENSG00000102316	1.856.335.604	-0.617163642	0.163089729	-3.784.196.875	0.000154206	0.005424068	10916	MAGED2
ENSG00000145220	1.701.110.917	-0.590463299	0.102022934	-5.787.554.554	7,14E-04	1,80E-01	55646	LYAR
ENSG00000082146	2.286.346.596	-0.579714501	0.145310771	-39.894.806	6,62E+00	0.002945294	55437	STRADB
ENSG00000225783	5.035.365.604	-0.573418164	0.145051355	-3.953.207.906	7,71E+00	0.003270242	440823	MIAT
ENSG00000189067	4.737.035.679	-0.56838916	0.153266148	-3.708.510.758	0.000208482	0.006841021	9516	LITAF
ENSG00000167107	6.825.988.122	-0.555441311	0.144377428	-3.847.147.851	0.000119501	0.004493294	80221	ACSF2
ENSG00000082898	1.113.136.207	-0.553510862	0.146392121	-3.781.015.393	0.00015619	0.005464403	7514	XPO1
ENSG00000129467	5.752.072.365	-0.549908574	0.117725504	-467.110.827	3,00E-01	0.00026171	196883	ADCY4
ENSG00000162148	1.840.144.431	-0.54391079	0.113898415	-4.775.402.629	1,79E-01	0.000179213	220004	PPP1R32
ENSG00000169180	4.330.355.121	-0.533366009	0.125352747	-4.254.920.776	2,09E+00	0.001210673	23214	XPO6
ENSG00000123352	3.465.470.015	-0.532992538	0.122369173	-4.355.611.188	1,33E+00	0.000856595	65244	SPATS2
ENSG00000165704	9.469.304.149	-0.525671898	0.141436923	-3.716.652.527	0.00020188	0.006663403	3251	HPRT1
ENSG00000125772	4.130.960.091	-0.520846218	0.136430448	-3.817.668.458	0.000134719	0.004925846	56261	GPCPD1
ENSG00000124659	1.502.320.007	-0.518772327	0.093824281	-5.529.190.519	3,22E-03	6,52E-01	6903	TBCC

ENSG00000273237	119.825.513	-0.513843278	0.128207403	-4.007.906.444	6,13E+00	0.002796246	NA	NA
ENSG00000271361	1.108.612.922	-0.51147837	0.137755367	-371.294.696	0.00020486	0.006746501	NA	HTATSF1P2
ENSG00000169057	4.979.882.918	-0.51072518	0.106793763	-4.782.350.274	1,73E-01	0.000174321	4204	MECP2
ENSG00000151065	7.041.437.824	-0.510251885	0.125302819	-4.072.150.083	4,66E+00	0.002257715	196513	DCP1B
ENSG00000272341	4.481.839.296	-0.508711606	0.131987289	-3.854.246.941	0.000116086	0.004432079	NA	
ENSG00000177426	1.021.552.777	-0.496117942	0.116057181	-4.274.771.628	1,91E+00	0.001130106	7050	TGIF1
ENSG00000137337	2.794.822.783	-0.495319281	0.124749887	-3.970.498.844	7,17E+00	0.003132804	9656	MDC1
ENSG00000110514	3.538.457.353	-0.492709823	0.122678895	-4.016.255.802	5,91E+00	0.002729901	8567	MADD
ENSG00000138002	4.686.992.823	-0.488391076	0.127050398	-3.844.073.553	0.000121009	0.004538293	26160	IFT172
ENSG00000069493	4.738.438.029	-0.48325734	0.068524648	-7.052.314.035	1,76E-07	1,83E-05	29121	CLEC2D
ENSG00000155592	156.172.641	-0.482625239	0.134010843	-360.138.946	0.000316521	0.009328737	342357	ZKSCAN2
ENSG00000126870	528.465.876	-0.481460715	0.09641396	-4.993.682.617	5,92E-02	6,80E+00	55112	WDR60
ENSG00000170584	4.714.432.756	-0.470288272	0.124281555	-378.405.527	0.000154294	0.005424068	134492	NUDCD2
ENSG00000047579	3.088.933.744	-0.469036354	0.087700389	-5.348.167.325	8,88E-03	1,49E+00	84062	DTNBP1
ENSG00000179144	432.874.284	-0.46837622	0.129051801	-3.629.365.998	0.000284118	0.008635417	168537	GIMAP7
ENSG00000151882	3.625.236.588	-0.463078784	0.127548441	-3.630.611.088	0.000282751	0.008635417	56477	CCL28
ENSG00000242247	180.548.378	-0.455092722	0.095622306	-475.927.364	1,94E-01	0.000191521	26286	ARFGAP3
ENSG00000121579	4.000.967.728	-0.452390755	0.121715223	-3.716.796.845	0.000201765	0.006663403	80218	NAA50
ENSG00000171262	6.580.076.703	-0.445493699	0.083227783	-535.270.417	8,66E-03	1,47E+00	283742	FAM98B
ENSG00000198585	6.303.842.422	-0.444195464	0.106915914	-4.154.624.412	3,26E+00	0.001671004	131870	NUDT16
ENSG00000178977	3.323.592.672	-0.439912544	0.123081066	-357.416.912	0.000351342	0.009991668	284029	LINC00324
ENSG00000187091	1.067.560.905	-0.434304739	0.118544778	-366.363.452	0.000248662	0.007835255	5333	PLCD1
ENSG00000213443	8.728.394.715	-0.433888421	0.067312739	-644.585.899	1,15E-05	6,21E-05	NA	
ENSG00000100802	266.795.932	-0.429696141	0.099556437	-4.316.106.075	1,59E+00	0.00098647	60686	C14orf93
ENSG00000139278	9.114.038.965	-0.426458752	0.094801784	-4.498.425.391	6,85E-01	0.000509563	11010	GLIPR1
ENSG00000204536	5.957.901.185	-0.414241559	0.112664573	-3.676.768.559	0.000236207	0.007574465	54535	CCHCR1
ENSG00000178904	1.037.352.094	-0.40903025	0.109596583	-373.214.419	0.000189857	0.006368694	147991	DPY19L3
ENSG00000243943	8.580.110.623	-0.404956959	0.092597401	-437.330.807	1,22E+00	0.000804358	84450	ZNF512
ENSG00000100429	2.379.719.287	-0.403230849	0.095069759	-424.142.076	2,22E+00	0.00126729	83933	HDAC10

ENSG00000244879	1.289.951.951	-0.398702536	0.099486123	-4.007.619.605	6,13E+00	0.002796246	NA	GABPB1-AS1
ENSG00000137274	1.536.925.198	-0.395973641	0.106780803	-3.708.284.906	0.000208668	0.006841021	670	BPHL
ENSG00000143952	1.438.164.571	-0.395298417	0.088633191	-4.459.936.676	8,20E-01	0.000580613	51542	VPS54
ENSG00000078304	7.817.482.446	-0.376006205	0.073549896	-5.112.260.182	3,18E-02	4,40E+00	5527	PPP2R5C
ENSG00000133574	676.408.043	-0.375658239	0.097750116	-3.843.046.474	0.000121516	0.00454565	55303	GIMAP4
ENSG00000115956	4.058.774.847	-0.370832896	0.092629432	-4.003.402.477	6,24E+00	0.002831662	5341	PLEK
ENSG00000204272	3.924.133.517	-0.370416855	0.103297209	-3.585.932.843	0.000335875	0.009628615	550643	NBDY
ENSG00000113119	4.244.571.395	-0.366421365	0.071602248	-511.745.614	3,10E-02	4,36E+00	55374	TMCO6
ENSG00000171448	8.495.728.625	-0.357145394	0.085803793	-4.162.349.727	3,15E+00	0.001633041	57684	ZBTB26
ENSG00000140265	1.439.269.283	-0.35660942	0.09527383	-3.742.994.462	0.00018184	0.006169459	146050	ZSCAN29
ENSG00000143303	7.995.309.408	-0.355200687	0.083144826	-4.272.072.027	1,94E+00	0.001139266	51093	RRNAD1
ENSG00000168397	2.780.165.075	-0.354233405	0.077387564	-4.577.394.455	4,71E-01	0.000381586	23192	ATG4B
ENSG00000158793	7.472.759.934	-0.351156801	0.075448068	-4.654.284.858	3,25E-01	0.00028065	4817	NIT1
ENSG00000133606	2.035.098.408	-0.34495929	0.091575733	-376.692.904	0.000165268	0.005735438	23608	MKRN1
ENSG00000171492	9.228.194.253	-0.341464018	0.088168315	-3.872.865.409	0.000107563	0.004195829	55144	LRRC8D
ENSG00000164483	4.735.806.008	-0.334558309	0.075432369	-4.435.208.848	9,20E-01	0.00063902	154075	SAMD3
ENSG00000133703	2.594.431.734	-0.326950329	0.081112161	-4.030.842.275	5,56E+00	0.002598775	3845	KRAS
ENSG00000123130	1.037.583.367	-0.319158062	0.088485648	-3.606.890.736	0.000309888	0.009151735	23597	ACOT9
ENSG00000124587	1.324.561.335	-0.317072351	0.074582728	-4.251.283.887	2,13E+00	0.001220815	5190	PEX6
ENSG00000115947	706.583.162	-0.313995161	0.053663004	-5.851.240.876	4,88E-04	1,29E-01	5000	ORC4
ENSG00000116406	3.168.784.562	-0.312874903	0.070004311	-4.469.366.247	7,85E-01	0.0005666	80267	EDEM3
ENSG00000213015	8.867.732.966	-0.308657475	0.084580737	-3.649.264.433	0.000262992	0.008180798	51157	ZNF580
ENSG00000263753	157.549.029	-0.308067676	0.070443311	-4.373.270.801	1,22E+00	0.000804358	339290	LINC00667
ENSG00000225697	1.725.946.966	-0.305721652	0.077213604	-3.959.427.302	7,51E+00	0.003230997	65010	SLC26A6
ENSG00000122484	1.944.702.127	-0.294992531	0.07047306	-4.185.890.742	2,84E-01	0.001517951	79871	RPAP2
ENSG00000271122	39.077.062	-0.294511592	0.081483479	-3.614.371.843	0.000301077	0.009057201	1,02E+08	
ENSG00000156469	3.293.640.265	-0.288190986	0.076512964	-3.766.564.126	0.00016551	0.005735438	51001	MTERF3
ENSG00000116273	4.514.737.188	-0.286874133	0.074360696	-3.857.873.139	0.000114378	0.004391212	148479	PHF13
ENSG00000002016	5.733.610.878	-0.281284735	0.055551885	-5.063.459.792	4,12E-02	5,13E+00	5893	RAD52

ENSG00000172992	4.182.211.864	-0.272825846	0.068758938	-3.967.860.094	7,25E+00	0.00315823	79877	DCAKD
ENSG00000171720	1.566.542.241	-0.267517724	0.069456073	-3.851.610.255	0.000117344	0.004446563	8841	HDAC3
ENSG00000121104	1.850.106.562	-0.25905742	0.063366561	-4.088.235.435	4,35E+00	0.002135136	81558	FAM117A
ENSG00000131051	1.228.643.637	-0.258669672	0.068145489	-3.795.844.391	0.000147142	0.005248536	9584	RBM39
ENSG00000100650	1.273.502.803	-0.257605178	0.058158702	-4.429.348.862	9,45E-02	0.000650436	6430	SRSF5
ENSG00000125352	7.705.706.129	-0.241885274	0.064189098	-3.768.323.324	0.000164348	0.005722964	7737	RNF113A
ENSG00000168918	7.746.884.172	-0.239572996	0.053107286	-451.111.353	6,45E-01	0.000482471	3635	INPP5D
ENSG00000133858	5.840.486.861	-0.23394613	0.058792218	-3.979.202.347	6,91E+00	0.00305174	196441	ZFC3H1
ENSG00000139218	4.793.298.943	-0.231865253	0.040611279	-5.709.380.661	1,13E-03	2,63E-01	9169	SCAF11
ENSG00000081791	1.794.202.522	-0.228577954	0.048770598	-4.686.798.279	2,78E-01	0.000248382	9812	DELE1
ENSG00000151502	2.761.685.044	-0.217841917	0.047097102	-4.625.378.339	3,74E-01	0.000313509	112936	VPS26B
ENSG00000143374	8.163.074.274	-0.216441456	0.05842106	-3.704.853.282	0.000211513	0.006887868	80222	TARS2
ENSG00000143486	8.895.245.258	-0.209978151	0.052590772	-3.992.680.527	6,53E+00	0.002923643	1939	EIF2D
ENSG00000166164	1.348.264.234	-0.191168816	0.052962812	-3.609.491.418	0.000306798	0.009111102	29117	BRD7
ENSG00000143379	146.954.318	-0.174873285	0.046145782	-3.789.583.321	0.0001509	0.005356406	9869	SETDB1
ENSG00000183283	7.000.559.028	0.153529037	0.038585933	397.888.622	6,92E+00	0.00305174	9802	DAZAP2
ENSG00000112308	6.748.540.846	0.158008512	0.041202295	3.834.944.456	0.000125593	0.004662268	81688	C6orf62
ENSG00000100393	4.229.053.025	0.165883401	0.042863488	3.870.039.716	0.000108818	0.004233441	2033	EP300
ENSG00000137075	2.032.657.774	0.16671348	0.042135233	3.956.628.859	7,60E+00	0.003233182	152006	RNF38
ENSG00000160551	4.789.306.684	0.184795073	0.048581109	3.803.846.349	0.000142467	0.005149774	57551	TAOK1
ENSG00000125484	8.033.065.159	0.18940068	0.048430322	3.910.787.101	9,20E+00	0.003728131	9329	GTF3C4
ENSG00000159346	136.980.951	0.190783344	0.052327755	3.645.930.238	0.000266426	0.008234942	51094	ADIPOR1
ENSG00000167548	1.085.524.537	0.190985207	0.050923138	3.750.460.268	0.00017651	0.006030699	8085	KMT2D
ENSG00000006114	7.862.367.596	0.19238896	0.05325988	3.612.267.991	0.000303531	0.009057201	NA	NA
ENSG00000144580	1.492.678.688	0.202004722	0.052807956	3.825.270.597	0.000130629	0.004812474	9125	CNOT9
ENSG00000229117	1.632.431.046	0.203756491	0.056456343	360.909.831	0.000307263	0.009111102	6171	RPL41
ENSG00000198851	1.342.101.554	0.206477709	0.04738732	4.357.235.415	1,32E+00	0.000854044	916	CD3E
ENSG00000166272	1.177.918.848	0.206508262	0.040062351	5.154.671.592	2,54E-02	3,74E+00	1,03E+08	WBP1L
ENSG00000071553	1.764.236.338	0.211179585	0.04567569	4.623.456.892	3,77E-01	0.00031462	537	ATP6AP1

ENSG00000132549	3.773.595.509	0.213571379	0.056939451	3.750.850.689	0.000176236	0.006030699	157680	VPS13B
ENSG00000068912	1.203.087.919	0.220575799	0.047312632	4.662.091.117	3,13E-01	0.000271818	27248	ERLEC1
ENSG00000204516	6.713.274.544	0.222925557	0.061990233	3.596.139.987	0.000322974	0.009425673	4277	MICB
ENSG00000114316	2.181.289.368	0.223937024	0.049053761	4.565.134.654	4,99E-01	0.000397948	1,08E+08	USP4
ENSG00000084463	1.541.439.208	0.224864347	0.053415225	420.974.255	2,56E+00	0.001396948	51729	WBP11
ENSG00000169826	2.044.588.288	0.225892151	0.046259432	4.883.158.763	1,04E-03	0.000111174	55454	CSGALNACT2
ENSG00000092841	6.431.453.666	0.228473317	0.060784818	3.758.723.401	0.000170783	0.005876288	4637	MYL6
ENSG00000182934	362.245.615	0.229618491	0.049495912	4.639.140.494	3,50E-01	0.000300243	6734	SRPRA
ENSG00000131023	1.986.332.439	0.230890963	0.059608855	3.873.433.963	0.000107312	0.004195829	9113	LATS1
ENSG00000082701	1.758.530.864	0.238514187	0.060147228	3.965.505.886	7,32E+00	0.003169357	2932	GSK3B
ENSG00000107771	3.467.062.168	0.239599817	0.065978936	3.631.459.247	0.000281823	0.008635417	54462	CCSER2
ENSG00000084093	2.214.325.876	0.239651971	0.064896233	3.692.848.711	0.000221756	0.007173387	5978	REST
ENSG00000152117	7.228.593.267	0.240653258	0.059736619	40.285.718	5,61E+00	0.002615613	150776	
ENSG00000080603	5.872.112.848	0.241739051	0.056998466	4.241.150.137	2,22E+00	0.00126729	10847	SRCAP
ENSG00000166794	3.792.994.798	0.244210854	0.064272244	3.799.631.668	0.000144911	0.005181645	5479	PPIB
ENSG00000104331	1.537.913.487	0.244458459	0.044836181	5.452.258.762	4,97E-04	9,07E-01	54928	IMPAD1
ENSG00000005302	2.602.869.267	0.246692232	0.064867128	3.803.039.208	0.000142932	0.005149774	10943	MSL3
ENSG00000167202	4.657.394.714	0.247744828	0.063855011	3.879.802.465	0.000104541	0.004110926	23102	TBC1D2B
ENSG00000076321	586.517.456	0.253199959	0.061098017	4.144.160.029	3,41E+00	0.00172769	27252	KLHL20
ENSG00000148396	4.271.520.608	0.253499449	0.060979684	4.157.113.182	3,22E+00	0.001661467	9919	SEC16A
ENSG00000106785	2.047.870.141	0.253854159	0.070122558	362.014.974	0.000294433	0.008909964	9830	TRIM14
ENSG00000197535	3.068.286.455	0.254640026	0.07015296	3.629.783.076	0.00028366	0.008635417	4644	MYO5A
ENSG00000105851	2.115.434.384	0.25512806	0.071147844	3.585.886.055	0.000335936	0.009628615	5294	PIK3CG
ENSG00000169032	1.766.778.869	0.257558091	0.061078666	4.216.825.776	2,48E+00	0.001368106	5604	MAP2K1
ENSG00000185418	8.467.761.835	0.257738933	0.068005041	378.999.745	0.000150649	0.005356406	123283	TARSL2
ENSG00000160654	4.721.431.004	0.265981569	0.066508278	399.922.502	6,36E+00	0.002861526	917	CD3G
ENSG00000118058	1.370.538.613	0.26618351	0.068347386	3.894.567.528	9,84E+00	0.003942801	4297	KMT2A
ENSG00000055609	7.738.171.056	0.270375616	0.058555596	4.617.417.203	3,89E-01	0.000322073	58508	KMT2C
ENSG00000169019	5.605.543.325	0.273844879	0.069755814	3.925.764.225	8,65E+00	0.003583208	54951	COMMD8

ENSG00000157106	1.549.524.276	0.280673922	0.073762757	3.805.089.906	0.000141753	0.005144353	23049	SMG1
ENSG00000047346	3.249.338.064	0.28587721	0.049687701	5.753.480.348	8,74E-04	2,13E-01	56204	FAM214A
ENSG00000072501	2.160.697.149	0.286857993	0.078605338	364.934.495	0.00026291	0.008180798	8243	SMC1A
ENSG00000138600	1.500.374.212	0.288026377	0.077990341	3.693.103.215	0.000221534	0.007173387	84888	SPPL2A
ENSG00000136816	7.840.208.563	0.289089325	0.067575892	4.277.994.958	1,89E+00	0.001118397	27348	TOR1B
ENSG00000141030	6.323.752.893	0.289128277	0.064894186	4.455.380.311	8,37E-01	0.00058738	8533	COPS3
ENSG00000135976	1.627.901.192	0.289251863	0.073645365	3.927.631.629	8,58E+00	0.003565638	375248	ANKRD36
ENSG00000102317	3.625.816.173	0.294243226	0.063966715	4.599.942.697	4,23E-01	0.000348329	5935	RBM3
ENSG00000055163	9.266.320.873	0.296430958	0.077726087	38.137.898	0.000136852	0.00499133	26999	CYFIP2
ENSG00000103591	96.479.502	0.298095611	0.082545882	3.611.271.738	0.000304699	0.009071952	79719	AAGAB
ENSG00000135749	275.899.192	0.299447526	0.076665444	3.905.899.588	9,39E+00	0.003793766	80003	PCNX2
ENSG00000102921	2.875.284.945	0.300716391	0.075695324	3.972.720.827	7,11E+00	0.003113035	9683	N4BP1
ENSG00000135439	2.434.416.893	0.304578688	0.076499239	3.981.460.385	6,85E+00	0.003037223	116986	AGAP2
ENSG00000127995	1.281.498.946	0.305364788	0.065844241	4.637.684.068	3,52E-01	0.000300597	64921	CASD1
ENSG00000198393	5.802.113.859	0.306795164	0.076542942	4.008.144.388	6,12E+00	0.002796246	7574	ZNF26
ENSG00000167395	1.401.064.519	0.307307629	0.0631089	4.869.481.597	1,12E-01	0.000118289	9726	ZNF646
ENSG00000133794	2.230.846.298	0.309523729	0.062620326	4.942.863.596	7,70E-02	8,38E+00	406	ARNTL
ENSG00000124333	10.040.144	0.31037662	0.071903329	4.316.582.085	1,58E+00	0.00098647	6845	VAMP7
ENSG00000134186	4.897.422.504	0.312442659	0.072391639	4.316.004.763	1,59E+00	0.00098647	55119	PRPF38B
ENSG00000173875	1.079.352.361	0.312686894	0.046115242	6.780.554.144	1,20E-06	1,03E-03	163049	ZNF791
ENSG00000152969	1.413.175.875	0.31404292	0.063621529	4.936.110.823	7,97E-02	8,55E+00	152789	JAKMIP1
ENSG00000167595	6.672.140.191	0.314858353	0.087475377	3.599.394.065	0.00031896	0.009360537	148137	PROSER3
ENSG00000175550	1.211.877.914	0.317255803	0.057644296	5.503.680.783	3,72E-03	7,33E-02	10589	DRAP1
ENSG00000116497	3.789.855.234	0.31816541	0.088692968	3.587.267.592	0.000334161	0.009615542	64766	S100PBP
ENSG00000198286	4.290.990.669	0.320410956	0.057823763	554.116.404	3,00E-03	6,26E-01	84433	CARD11
ENSG00000170348	3.782.697.432	0.320932161	0.076138979	4.215.083.594	2,50E+00	0.001369423	10972	TMED10
ENSG00000090020	7.823.946.309	0.32154282	0.071024757	4.527.193.521	5,98E-01	0.000451822	6548	SLC9A1
ENSG00000148248	4.768.829.527	0.322152293	0.0649797	4.957.737.479	7,13E-02	7,94E+00	6836	SURF4
ENSG00000184887	7.372.780.784	0.322571418	0.081356852	3.964.895.499	7,34E+00	0.003169357	90135	BTBD6

ENSG00000170113	1.622.525.873	0.325227106	0.069196836	4.700.028.592	2,60E-01	0.000241673	123606	NIPA1
ENSG00000163513	4.034.860.847	0.325345282	0.090400469	3.598.933.561	0.000319525	0.009360537	7048	TGFBR2
ENSG00000236287	2.532.375.832	0.327574373	0.09113137	3.594.529.232	0.000324979	0.009425673	58486	ZBED5
ENSG00000120800	5.785.346.365	0.329481469	0.087039235	378.543.616	0.000153439	0.005420157	27340	UTP20
ENSG00000044574	543.046.755	0.329517148	0.084208919	3.913.090.806	9,11E+00	0.003703015	3309	HSPA5
ENSG00000145734	3.155.815.568	0.330846746	0.065275857	5.068.439.707	4,01E-03	5,04E-01	55814	BDP1
ENSG00000142039	1.227.678.329	0.330938634	0.08383249	3.947.617.844	7,89E+00	0.003318584	90324	CCDC97
ENSG00000149600	1.111.161.638	0.333683654	0.089544688	3.726.448.338	0.000194197	0.006483153	149951	COMMD7
ENSG00000144746	6.111.810.865	0.333699542	0.087775018	3.801.759.881	0.000143672	0.00514995	10550	ARL6IP5
ENSG00000124767	2.729.590.185	0.335080851	0.079851363	419.630.722	2,71E+00	0.001460527	2739	GLO1
ENSG00000073050	5.574.495.734	0.335918966	0.088330262	3.802.988.444	0.000142961	0.005149774	7515	XRCC1
ENSG00000160299	2.237.909.082	0.338471412	0.083334134	4.061.617.927	4,87E+00	0.002338741	5116	PCNT
ENSG00000137845	2.988.908.949	0.339685832	0.062406436	5.443.121.802	5,24E-03	9,39E-01	102	ADAM10
ENSG00000002834	857.151.978	0.339926349	0.094559084	3.594.856.629	0.00032457	0.009425673	3927	LASP1
ENSG00000065613	2.965.322.468	0.342746649	0.036439166	9.405.995.937	5,15E-16	2,51E-12	9748	SLK
ENSG00000111615	8.161.388.264	0.34330414	0.076382686	4.494.528.269	6,97E-01	0.000516349	11103	KRR1
ENSG00000137449	9.251.953.358	0.343607613	0.077597419	4.428.080.441	9,51E-01	0.0006512	132864	CPEB2
ENSG00000143337	234.129.112	0.34385595	0.057908741	59.378.937	2,89E-04	8,26E-02	26092	TOR1AIP1
ENSG00000119392	1.349.038.184	0.344398226	0.095971866	3.588.533.203	0.000332544	0.009587904	2733	GLE1
ENSG00000198843	1.468.156.843	0.345380227	0.06862226	503.306.402	4,83E-02	5,87E+00	51714	SELENOT
ENSG00000116199	1.018.182.843	0.346031075	0.082755082	4.181.387.609	2,90E+00	0.001537073	9917	FAM20B
ENSG00000143384	1.718.796.672	0.347035161	0.05780514	6.003.534.612	1,93E-04	5,75E-02	4170	MCL1
ENSG00000113013	4.049.404.333	0.347273867	0.070302174	4.939.731.573	7,82E-02	8,45E+00	3313	HSPA9
ENSG00000160404	4.016.001.563	0.348838551	0.081289516	4.291.310.457	1,78E+00	0.001066389	27433	TOR2A
ENSG00000134287	2.612.193.984	0.348937808	0.077979701	4.474.726.183	7,65E-01	0.000555832	377	ARF3
ENSG00000102580	1.664.860.081	0.349525133	0.074759739	4.675.312.373	2,94E-01	0.000259513	5611	DNAJC3
ENSG00000247556	5.760.779.793	0.352263036	0.064586612	5.454.118.545	4,92E-03	9,07E-01	729082	OIP5-AS1
ENSG00000067955	2.766.985.832	0.358231644	0.071821375	4.987.813.792	6,11E-02	6,96E+00	865	CBFB
ENSG00000149177	4.040.145.973	0.358353038	0.07837139	4.572.498.206	4,82E-01	0.000388457	5795	PTPRJ

ENSG00000145817	8.198.297.778	0.360636771	0.088876441	4.057.731.899	4,96E+00	0.002370191	81555	YIPF5
ENSG00000146112	4.614.243.375	0.361873236	0.061357433	5.897.789.731	3,68E-04	1,03E-01	1,08E+08	PPP1R18
ENSG00000136819	208.004.839	0.363184123	0.085795784	4.233.123.181	2,30E+00	0.001288238	51759	C9orf78
ENSG00000146066	9.283.972.288	0.364298211	0.060768619	5.994.841.034	2,04E-04	5,94E-02	192286	HIGD2A
ENSG00000122188	1.531.757.347	0.364819963	0.094130602	3.875.678.631	0.000106328	0.004169942	54900	LAX1
ENSG00000127526	1.328.504.902	0.366926492	0.086048052	4.264.204.523	2,01E+00	0.001170713	79939	SLC35E1
ENSG00000104695	7.197.718.927	0.370137201	0.082964305	4.461.403.027	8,14E-01	0.000579466	5516	PPP2CB
ENSG00000114841	2.971.763.727	0.374919549	0.104664971	3.582.091.957	0.000340854	0.009750423	25981	DNAH1
ENSG00000171130	8.505.356.271	0.378818665	0.1050146	360.729.522	0.000309406	0.009151735	155066	ATP6V0E2
ENSG00000141429	1.492.395.929	0.383065239	0.098460656	3.890.541.216	0.000100021	0.003994598	2589	GALNT1
ENSG00000189043	9.221.542.109	0.384760079	0.095290339	4.037.765.855	5,40E+00	0.002547772	4697	NDUFA4
ENSG00000198937	2.255.446.965	0.387202299	0.099787595	3.880.264.873	0.000104343	0.004110926	154467	CCDC167
ENSG00000127946	139.812.061	0.391000615	0.107644341	363.233.785	0.000280865	0.008635417	3092	HIP1
ENSG00000108826	3.190.436.861	0.394438419	0.105528079	3.737.757.968	0.000185669	0.006270182	51264	MRPL27
ENSG00000152022	1.665.579.954	0.395774634	0.071771529	5.514.368.197	3,50E-03	7,00E-01	NA	NA
ENSG00000131871	6.553.742.837	0.396338054	0.062819353	6.309.171.264	2,81E-05	1,24E-02	55829	SELENOS
ENSG00000108582	2.307.296.007	0.39737302	0.069218174	5.740.876.987	9,42E-04	2,25E-01	1362	CPD
ENSG00000205045	5.011.793.137	0.39797751	0.097902675	4.065.032.028	4,80E+00	0.002312374	1,01E+08	SLFN12L
ENSG00000149428	2.831.217.194	0.404784707	0.105119832	3.850.697.815	0.000117782	0.004451601	10525	HYOU1
ENSG00000043462	8.795.681.771	0.405965494	0.06547655	620.016.627	5,64E-05	2,18E-02	3937	LCP2
ENSG00000166446	1.203.847.993	0.407495643	0.074435069	5.474.511.504	4,39E-04	8,31E-01	124359	CDYL2
ENSG00000107897	1.652.726.301	0.416957238	0.07729755	5.394.184.409	6,88E-03	1,18E+00	91452	ACBD5
ENSG00000167977	8.319.181.873	0.418056789	0.111552037	3.747.639.213	0.000178507	0.006070478	54442	KCTD5
ENSG00000099331	9.144.392.658	0.419122109	0.098860531	4.239.529.228	2,24E+00	0.001271508	4650	MYO9B
ENSG00000136878	4.039.220.794	0.420202419	0.080941021	5.191.464.266	2,09E-02	3,20E+00	10868	USP20
ENSG00000179119	120.569.501	0.420999969	0.086865573	4.846.568.722	1,26E-01	0.000131842	144108	SPTY2D1
ENSG00000172071	1.107.181.356	0.423960409	0.114465617	3.703.823.193	0.000212374	0.006900509	9451	EIF2AK3
ENSG00000135932	5.071.786.769	0.425533871	0.09873893	4.309.686.876	1,63E+00	0.001006369	51719	CAB39
ENSG00000111860	2.294.644.228	0.427619495	0.080328852	5.323.361.239	1,02E-02	1,69E-01	387119	CEP85L

ENSG00000221963	5.928.979.037	0.428842601	0.104190123	411.596.214	3,86E+00	0.001913281	80830	APOL6
ENSG00000137996	143.625.985	0.433714567	0.098805245	4.389.590.526	1,14E+00	0.000759995	8634	RTCA
ENSG00000131323	1.667.351.628	0.435180666	0.083371671	5.219.766.613	1,79E-02	2,78E+00	7187	TRAF3
ENSG00000213246	744.178.303	0.44734692	0.102942194	4.345.612.861	1,39E+00	0.000892611	6827	SUPT4H1
ENSG00000156502	8.442.441.293	0.451380806	0.088568406	5.096.408.835	3,46E-02	4,59E+00	6832	SUPV3L1
ENSG00000177409	7.138.475.155	0.455145533	0.124471636	3.656.620.492	0.000255562	0.008018061	219285	SAMD9L
ENSG00000076641	7.867.264.967	0.457214826	0.119762067	3.817.693.192	0.000134705	0.004925846	55824	PAG1
ENSG00000136003	1.480.428.848	0.460729452	0.128658255	3.581.032.961	0.000342238	0.009751791	23479	ISCU
ENSG00000163600	1.178.043.752	0.461131313	0.116500617	3.958.187.731	7,55E+00	0.003230997	29851	ICOS
ENSG00000267554	8.693.930.283	0.462154844	0.11904998	3.882.023.687	0.000103591	0.004095626	NA	
ENSG00000081087	1.456.730.021	0.467289895	0.096605406	4.837.098.826	1,32E-01	0.000136317	28962	OSTM1
ENSG00000149929	3.220.486.951	0.467753549	0.092997379	5.029.749.809	4,91E-03	5,92E+00	8479	HIRIP3
ENSG00000167378	4.988.472.577	0.468994942	0.093488507	5.016.605.305	5,26E-02	6,19E+00	126298	IRGQ
ENSG00000155926	3.018.698.839	0.47088641	0.112187231	4.197.326.245	2,70E+00	0.001459358	6503	SLA
ENSG00000105287	2.482.734.915	0.474336978	0.087310815	5.432.740.245	5,55E-03	9,75E-01	25865	PRKD2
ENSG00000088543	7.418.459.846	0.476415229	0.123647289	3.853.017.992	0.000116671	0.004432579	51161	C3orf18
ENSG00000127603	3.352.823.841	0.483416068	0.106162394	4.553.552.791	5,27E-01	0.000413727	643314	MACF1
ENSG00000176022	265.639.075	0.483933872	0.120887602	400.317.207	6,25E+00	0.002831662	126792	B3GALT6
ENSG00000254995	2.945.001.763	0.485645418	0.114943851	4.225.066.514	2,39E-01	0.001330106	NA	STX16-NPEPL1
ENSG00000153283	1.404.325.141	0.486425392	0.076087548	6.392.969.739	1,63E-05	7,66E-03	10225	CD96
ENSG00000156675	1.794.037.819	0.490526066	0.112218869	4.371.154.963	1,24E+00	0.000808551	80223	RAB11FIP1
ENSG00000132383	2.626.574.259	0.495958544	0.098194036	5.050.801.093	4,40E-02	5,39E+00	6117	RPA1
ENSG00000106799	3.221.316.768	0.49662344	0.118387694	4.194.890.734	2,73E+00	0.001464281	7046	TGFBR1
ENSG00000169410	1.123.639.019	0.499128736	0.103156378	4.838.564.014	1,31E-01	0.000136283	5780	PTPN9
ENSG00000174799	6.584.936.357	0.499767061	0.118879217	4.203.990.178	2,62E-01	0.001423515	9662	CEP135
ENSG00000013725	5.824.049.197	0.501294154	0.129850865	3.860.537.662	0.000113138	0.004355059	923	CD6
ENSG00000108219	4.899.469.798	0.502059116	0.134867035	3.722.622.922	0.000197164	0.006552218	81619	TSPAN14
ENSG00000168237	3.422.077.611	0.510632245	0.130893982	390.111.322	9,58E+00	0.003858885	132158	GLYCTK
ENSG00000107960	7.984.501.749	0.513857055	0.083002686	6.190.848.497	5,98E-05	2,18E-02	79991	STN1

ENSG00000115904	1.221.876.262	0.514404792	0.104051209	494.376.564	7,66E-02	8,38E+00	6654	SOS1
ENSG00000145246	1.087.875.088	0.516858927	0.14234856	3.630.938.935	0.000282392	0.008635417	57205	ATP10D
ENSG00000163297	5.287.107.893	0.522303347	0.102779025	5.081.808.748	3,74E-02	4,83E+00	118429	ANTXR2
ENSG00000135272	4.902.601.151	0.522866051	0.120926114	4.323.847.301	1,53E+00	0.000971015	29969	MDFIC
ENSG00000203739	1.361.475.181	0.526307284	0.138301516	3.805.506.242	0.000141514	0.005144353	1,02E+08	
ENSG00000233355	1.711.040.698	0.529964325	0.137987277	3.840.675.291	0.000122696	0.004578048	1,01E+08	CHRM3-AS2
ENSG00000164938	3.995.891.085	0.535861725	0.139853907	3.831.582.078	0.000127322	0.004714464	94241	TP53INP1
ENSG00000099326	8.483.239.448	0.537906028	0.148854516	361.363.593	0.000301933	0.009057201	7593	MZF1
ENSG00000163584	3.156.553.486	0.540362932	0.106976803	5.051.215.956	4,39E-02	5,39E+00	200916	RPL22L1
ENSG00000100647	2.818.883.933	0.541921423	0.088100272	6.151.188.962	7,69E-05	2,61E-03	9766	SUSD6
ENSG00000163376	223.371.706	0.54302737	0.12415143	4.373.911.522	1,22E-01	0.000804358	84541	KBTBD8
ENSG00000090339	8.543.112.218	0.546606877	0.132067472	4.138.845.595	3,49E+00	0.001756001	3383	ICAM1
ENSG00000106351	9.752.107.207	0.550834018	0.108333476	5.084.614.998	3,68E-02	4,82E+00	3268	AGFG2
ENSG00000105810	6.393.450.699	0.556325945	0.11068861	5.026.045.101	5,01E-02	5,99E+00	1021	CDK6
ENSG00000105339	2.019.278.784	0.556957651	0.098103172	567.726.445	1,37E-03	3,03E-01	22898	DENND3
ENSG00000121858	2.491.590.658	0.557523411	0.121344059	4.594.567.025	4,34E-01	0.000355421	8743	TNFSF10
ENSG00000052802	5.805.433.332	0.566064283	0.094039889	6.019.406.131	1,75E-04	5,32E-02	6307	MSMO1
ENSG00000117984	2.456.571.906	0.566793143	0.12504488	4.532.717.737	5,82E-01	0.000447111	1509	CTSD
ENSG00000186810	2.090.945.388	0.568343714	0.143317417	3.965.629.069	7,32E+00	0.003169357	2833	CXCR3
ENSG00000154229	3.577.370.245	0.571046408	0.138614319	4.119.678.353	3,79E+00	0.001889109	5578	PRKCA
ENSG00000144597	165.053.872	0.574511628	0.133759493	429.510.919	1,75E+00	0.001052623	85403	EAF1
ENSG00000108798	130.953.687	0.575881193	0.122765418	4.690.907.265	2,72E-01	0.000247085	51225	ABI3
ENSG00000101445	4.847.422.361	0.582124485	0.095370644	6.103.812.056	1,04E-04	3,36E-02	26051	PPP1R16B
ENSG00000100365	2.666.120.069	0.589288492	0.129169929	4.562.118.278	5,06E-01	0.000401515	4689	NCF4
ENSG00000136156	9.409.249.776	0.591789556	0.14077504	4.203.796.036	2,62E+00	0.001423515	9445	ITM2B
ENSG00000114738	2.102.386.567	0.591864173	0.098320618	601.973.609	1,75E-04	5,32E-02	7867	MAPKAPK3
ENSG00000136867	8.745.227.897	0.592795222	0.141743649	4.182.164.242	2,89E+00	0.001537073	1318	SLC31A2
ENSG00000163754	3.176.499.139	0.603450656	0.082396502	7.323.741.188	2,41E-08	2,93E-06	2992	GYG1
ENSG00000198879	3.247.199.471	0.618958276	0.142530704	4.342.631.162	1,41E+00	0.000900848	57713	SFMBT2

ENSG00000148400	1.906.622.067	0.624772004	0.171178504	3.649.827.463	0.000262417	0.008180798	4851	NOTCH1
ENSG00000196937	6.932.148.053	0.631161553	0.143079717	441.125.803	1,03E+00	0.000698263	10447	FAM3C
ENSG00000185650	2.907.026.596	0.639455172	0.16618823	3.847.776.532	0.000119195	0.004493294	677	ZFP36L1
ENSG00000173208	1.455.622.391	0.642640424	0.099886339	643.371.687	1,25E-05	6,49E-03	225	ABCD2
ENSG00000054148	1.049.179.561	0.647369151	0.179216258	3.612.223.338	0.000303583	0.009057201	29085	PHPT1
ENSG00000138658	3.829.162.831	0.648816534	0.148904594	4.357.263.373	1,32E+00	0.000854044	55345	ZGRF1
ENSG00000110025	1.112.787.753	0.653371558	0.146617008	44.563.149	8,34E-01	0.00058738	29907	SNX15
ENSG00000118971	6.669.297.429	0.653668205	0.161604571	4.044.862.108	5,24E+00	0.002479842	894	CCND2
ENSG00000163935	3.915.218.887	0.660159719	0.130030894	5.076.945.158	3,84E-02	4,91E+00	51460	SFMBT1
ENSG00000273151	1.234.659.337	0.661180482	0.153847932	4.297.623.451	1,73E+00	0.001045076	NA	
ENSG00000229645	5.178.643.574	0.663728891	0.146581759	4.528.045.617	5,95E-01	0.000451822	NA	NA
ENSG00000102241	2.107.186.202	0.669801475	0.129364522	5.177.628.819	2,25E-02	3,35E-01	27336	HTATSF1
ENSG00000075420	6.887.931.567	0.672878394	0.17181047	3.916.399.244	8,99E+00	0.003683368	64778	FNDC3B
ENSG00000167106	3.407.841.942	0.674288559	0.143133163	4.710.917.761	2,47E-01	0.000233617	399665	FAM102A
ENSG00000239697	5.382.923.292	0.676222448	0.109396783	6.181.374.184	6,35E-06	2,26E-02	8742	TNFSF12
ENSG00000081320	6.201.859.568	0.677083308	0.133492569	5.072.067.398	3,94E-02	4,99E+00	9262	STK17B
ENSG00000067082	769.894.259	0.68345091	0.115970864	5.893.298.396	3,79E-04	1,04E-01	1316	KLF6
ENSG00000272498	100.087.537	0.690435843	0.184635407	3.739.455.255	0.000184419	0.006242449	NA	
ENSG00000164466	2.050.881.706	0.710100134	0.137848337	5.151.314.491	2,59E-02	3,77E+00	94081	SFXN1
ENSG00000136950	1.539.221.266	0.717185505	0.151822565	4.723.839.995	2,31E-01	0.000223601	81873	ARPC5L
ENSG00000105963	2.334.155.746	0.719128611	0.199733569	3.600.439.399	0.00031768	0.009344015	11033	ADAP1
ENSG00000244625	1.361.307.304	0.719557957	0.176771398	4.070.556.463	4,69E-01	0.00226569	NA	MIATNB
ENSG00000161791	3.423.413.449	0.721816509	0.128727455	5.607.323.696	2,05E-03	4,41E-01	91010	FMNL3
ENSG00000137628	1.980.109.291	0.731710466	0.199473881	3.668.201.884	0.000244262	0.00778066	55601	DDX60
ENSG00000172123	4.963.517.778	0.734237113	0.190528635	385.368.379	0.000116354	0.004432079	55106	SLFN12
ENSG00000156127	283.785.446	0.737447028	0.203506288	3.623.706.349	0.000290411	0.008808338	10538	BATF
ENSG00000038002	1.716.522.968	0.744225639	0.180623099	4.120.323.713	3,78E-01	0.001889109	175	AGA
ENSG00000111670	7.954.875.731	0.751589988	0.189124217	3.974.054.718	7,07E+00	0.003104966	79158	GNPTAB
ENSG00000176438	5.395.795.394	0.754338413	0.17518961	4.305.839.911	1,66E+00	0.001019718	161176	SYNE3

ENSG00000116663	3.547.520.259	0.760653574	0.170859793	4.451.916.749	8,51E-01	0.000594081	26270	FBXO6
ENSG00000198133	4.695.793.977	0.762278475	0.204599736	3.725.706.051	0.000194769	0.006487418	161145	TMEM229B
ENSG00000151692	207.711.947	0.763340754	0.160995912	4.741.367.304	2,12E-01	0.000206464	9781	RNF144A
ENSG00000272529	9.158.591.015	0.763654699	0.18865777	404.783.063	5,17E+00	0.002456585	NA	
ENSG00000126246	321.713.888	0.767791014	0.196193947	391.342.865	9,10E+00	0.003703015	79713	IGFLR1
ENSG00000127561	3.524.808.426	0.769769233	0.180791524	4.257.772.789	2,06E+00	0.001200097	9143	SYNGR3
ENSG00000196843	1.092.434.978	0.771326511	0.154131348	5.004.345.443	5,61E-02	6,49E+00	10865	ARID5A
ENSG00000123146	5.508.142.634	0.776947297	0.117641525	6.604.362.704	3,99E-06	2,43E-03	976	ADGRE5
ENSG00000105717	1.272.552.494	0.786626688	0.154131622	5.103.603.504	3,33E-02	4,50E+00	80714	PBX4
ENSG00000173706	1.200.683.804	0.80523572	0.171599326	4.692.534.284	2,70E-01	0.000247085	57493	HEG1
ENSG00000154814	1.882.126.235	0.806029025	0.116604424	6.912.508.106	4,76E-07	4,63E-04	92106	OXNAD1
ENSG00000267745	507.549.848	0.822472452	0.220980491	3.721.923.365	0.000197711	0.006555469	NA	
ENSG00000137193	8.195.850.929	0.842785192	0.158424683	5.319.784.621	1,04E-03	1,70E+00	5292	PIM1
ENSG00000125735	9.592.210.033	0.853953573	0.170199023	5.017.382.343	5,24E-02	6,19E+00	8740	TNFSF14
ENSG00000181215	1.149.248.307	0.85524556	0.233398135	3.664.320.458	0.000247996	0.007831202	389197	C4orf50
ENSG00000171552	1.780.202.522	0.856191325	0.136812672	6.258.128.812	3,90E-05	1,62E-02	598	BCL2L1
ENSG00000205593	2.032.591.403	0.856430607	0.221828211	3.860.783.104	0.000113024	0.004355059	414918	DENND6B
ENSG00000171522	2.429.889.909	0.860143958	0.168082012	5.117.406.371	3,10E-02	4,36E+00	5734	PTGER4
ENSG00000271964	128.828.534	0.864949278	0.225370223	3.837.903.993	0.000124089	0.004618199	NA	
ENSG00000117602	1.632.022.025	0.867148766	0.205305432	4.223.701.039	2,40E+00	0.001333106	11123	RCAN3
ENSG00000105486	751.827.423	0.884369057	0.212326709	416.513.336	3,11E+00	0.00162131	3978	LIG1
ENSG00000188157	1.940.449.169	0.887629508	0.243895996	3.639.377.128	0.000273298	0.00842949	375790	AGRN
ENSG00000147889	7.383.727.382	0.889937397	0.220663471	403.300.734	5,51E+00	0.002588678	1029	CDKN2A
ENSG00000130396	3.052.227.804	0.918707794	0.211709262	433.947.852	1,43E+00	0.000909877	4301	AFDN
ENSG00000260852	935.242.367	0.924513526	0.162180293	5.700.529.384	1,19E-03	2,72E-01	283932	FBXL19-AS1
ENSG00000183688	255.118.416	0.932541752	0.171369354	544.170.663	5,28E-03	9,39E-01	359845	RFLNB
ENSG00000237422	1.788.223.585	0.936863611	0.204462869	458.207.211	4,60E-01	0.000375232	NA	
ENSG00000120875	1.653.246.814	0.937002926	0.247778316	3.781.617.937	0.000155812	0.005464295	1846	DUSP4
ENSG00000134460	1.487.749.364	0.948396983	0.196981795	4.814.642.806	1,47E-01	0.000151503	3559	IL2RA

ENSG00000102471	1.498.902.921	0.953021531	0.259678202	3.670.009.738	0.000242541	0.007742744	54602	NDFIP2
ENSG00000175048	7.429.580.605	0.95757274	0.237463128	4.032.511.268	5,52E-01	0.002588678	79683	ZDHHC14
ENSG00000124145	3.366.990.073	0.957698446	0.183246572	5.226.283.027	1,73E-02	2,71E+00	6385	SDC4
ENSG00000230537	3.308.313.947	0.958425475	0.18750385	5.111.497.571	3,20E-02	4,40E+00	NA	
ENSG00000168675	6.741.728.027	0.962637009	0.258026428	3.730.769.042	0.000190896	0.006387578	753	LDLRAD4
ENSG00000111859	1.638.295.265	0.9636462	0.129491386	7.441.778.402	9,93E-09	1,51E-05	4739	NEDD9
ENSG00000116679	116.557.228	0.977665872	0.177778536	5.499.347.063	3,81E-05	7,42E-01	10625	IVNS1ABP
ENSG00000104081	1.698.638.684	0.982439202	0.252628189	3.888.874.018	0.00010071	0.004003443	90427	BMF
ENSG00000177674	6.529.458.923	0.983441511	0.263509369	3.732.093.149	0.000189895	0.006368694	57085	AGTRAP
ENSG00000134107	8.429.764.701	0.990420554	0.150738089	6.570.473.064	5,02E-06	2,93E-03	8553	BHLHE40
ENSG00000267519	6.645.196.964	1.013.396.077	0.153157455	661.669.442	3,67E-07	2,33E-03	NA	
ENSG00000177606	9.325.496.308	1.043.707.716	0.243424718	4.287.599.578	1,81E+00	0.001075503	3725	JUN
ENSG00000029993	7.621.115.745	1.045.218.382	0.256311716	4.077.918.869	4,54E+00	0.002209777	3149	HMGB3
ENSG00000233609	3.378.061.853	1.047.905.279	0.160953976	651.058.956	7,49E-06	4,20E-03	NA	RPL10P19
ENSG00000175305	9.488.412.288	1.065.345.878	0.278270284	382.845.722	0.000128949	0.004762627	9134	CCNE2
ENSG00000080546	1.723.200.142	1.080.003.462	0.259979016	4.154.194.745	3,26E+00	0.001671004	27244	SESN1
ENSG00000164484	9.700.807.166	1.114.188.397	0.284465868	3.916.773.583	8,97E-01	0.003683368	114801	TMEM200A
ENSG00000138166	1.535.765.406	1.142.086.163	0.168992967	6.758.187.534	1,40E-06	1,13E-04	1847	DUSP5
ENSG00000270069	7.164.785.087	1.226.591.109	0.232160616	5.283.372.915	1,27E-02	2,06E+00	NA	MIR222HG
ENSG00000144802	1.342.503.413	1.237.991.762	0.281485356	4.398.068.096	1,09E+00	0.000734282	64332	NFKBIZ
ENSG00000079385	5.988.002.696	1.266.294.171	0.296922213	4.264.733.711	2,00E+00	0.001170713	634	CEACAM1
ENSG00000099889	31.385.472	1.295.170.184	0.307192578	4.216.150.634	2,49E+00	0.001368106	421	ARVCF
ENSG00000227507	9.129.644.081	1.315.625.301	0.317241544	4.147.077.605	3,37E+00	0.001711772	4050	LTB
ENSG00000176658	7.463.220.146	1.342.608.683	0.374028393	3.589.590.281	0.000331198	0.009568018	4642	MYO1D
ENSG00000186187	2.067.440.408	1.352.871.941	0.183292811	7.380.932.906	1,57E-08	2,08E-05	84937	ZNRF1
ENSG00000114554	4.841.205.233	1.391.964.684	0.335281889	4.151.625.037	3,30E+00	0.001683973	5361	PLXNA1
ENSG00000184371	2.615.620.887	1.416.508.086	0.353945297	4.002.053.702	6,28E-01	0.002836275	1435	CSF1
ENSG00000227630	3.159.277.108	1.423.619.684	0.331850096	4.289.948.084	1,79E+00	0.001068554	1,01E+08	LINC01132
ENSG00000173114	1.457.775.225	1.505.712.165	0.341838945	4.404.741.433	1,06E-01	0.000715335	54674	LRRN3

ENSG00000101412	2.309.546.129	1.509.723.662	0.330448825	4.568.706.402	4,91E-01	0.000393377	1869	E2F1
ENSG00000160233	3.288.177.545	1.536.070.839	0.339199318	4.528.519.833	5,94E-01	0.000451822	81543	LRRC3
ENSG00000270426	7.801.869.602	1.566.432.828	0.39656837	3.949.969.156	7,82E+00	0.003295649	NA	
ENSG00000179862	2.350.078.085	1.573.511.463	0.390956395	4.024.774.846	5,70E+00	0.002641297	163732	CITED4
ENSG00000150938	187.554.169	1.575.528.817	0.286997484	5.489.695.569	4,03E-03	7,73E-01	51232	CRIM1
ENSG00000121297	1.702.389.716	1.594.128.755	0.38223649	4.170.530.017	3,04E+00	0.001594777	57616	TSHZ3
ENSG00000130164	2.801.353.608	1.635.456.649	0.256501493	6.376.012.213	1,82E-05	8,29E-03	3949	LDLR
ENSG00000140323	3.974.682.367	1.637.914.733	0.255461339	6.411.595.358	1,44E-05	7,24E-03	85455	DISP2
ENSG00000232810	172.193.783	1.724.553.085	0.194131623	8.883.421.758	6,48E-14	1,89E-10	7124	TNF
ENSG00000153094	3.129.067.083	173.181.912	0.302427179	5.726.400.413	1,03E-03	2,41E-01	10018	BCL2L11
ENSG00000114737	665.098.108	1.735.704.821	0.200039844	8.676.795.523	4,07E-13	8,48E-10	1154	CISH
ENSG00000129038	4.140.244.472	1.738.474.492	0.449305746	3.869.246.067	0.000109172	0.004235948	4016	LOXL1
ENSG00000166900	186.612.633	1.785.636.111	0.171382743	1.041.899.601	2,03E-20	1,48E-16	6809	STX3
ENSG00000269430	2.694.594.643	1.800.699.289	0.4183384	430.440.832	1,67E+00	0.001022039	NA	NA
ENSG00000139318	1.988.115.767	1.834.032.981	0.321881564	5.697.850.352	1,21E-03	2,72E-01	1848	DUSP6
ENSG00000185338	2.831.560.468	1.928.948.628	0.28838207	6.688.864.638	2,25E-06	1,49E-03	8651	SOCS1
ENSG00000273004	1.000.351.461	1.953.844.238	0.493785384	3.956.869.325	7,59E+00	0.003233182	NA	
ENSG00000116991	435.338.939	2.013.137.482	0.549982734	3.660.364.879	0.000251856	0.007918819	57568	SIPA1L2
ENSG00000254477	1.163.012.176	2.024.987.986	0.523950033	3.864.849.432	0.000111158	0.00430154	NA	
ENSG00000160293	5.840.208.352	2.046.293.686	0.401066398	5.102.131.961	3,36E-02	4,50E+00	7410	VAV2
ENSG00000143153	7.428.080.924	2.135.688.188	0.594549172	3.592.113.635	0.000328007	0.009494624	481	ATP1B1
ENSG00000136158	4.909.905.912	2.174.091.145	0.541790412	4.012.789.992	6,00E+00	0.002761569	10253	SPRY2
ENSG00000107282	4.524.666.264	2.254.945.381	0.303221033	7.436.639.081	1,03E-08	1,51E-05	320	APBA1
ENSG00000237372	1.237.036.579	2.256.487.252	0.487518283	462.851.821	3,68E-01	0.000310578	1E+08	
ENSG00000113070	1.055.565.992	2.260.510.095	0.474484507	4.764.138.898	1,90E-01	0.00018823	1839	HBEGF
ENSG00000186265	1.792.733.432	2.324.364.569	0.375269626	6.193.852.112	5,87E-05	2,18E-02	151888	BTLA
ENSG00000099282	3.212.361.619	2.354.744.948	0.520535385	4.523.698.132	6,08E-01	0.000456984	23555	TSPAN15
ENSG00000007237	2.008.966.647	2.383.921.189	0.64870402	3.674.898.126	0.000237944	0.007612653	8522	GAS7
ENSG00000161381	3.287.829.252	2.395.499.885	0.356019236	6.728.568.692	1,71E-07	1,25E-03	57125	PLXDC1

ENSG00000111537	176.722.336	24.350.288	0.393151183	6.193.619.408	5,88E-05	2,18E-02	3458	IFNG
ENSG00000099958	4.568.840.351	2.450.827.148	0.521232289	4.701.986.423	2,58E-01	0.000240945	91319	DERL3
ENSG00000061337	2.845.553.429	2.459.518.912	0.418816708	5.872.542.487	4,29E-04	1,16E-01	11178	LZTS1
ENSG00000182621	2.553.402.461	2.498.031.688	0.558278089	4.474.529.339	7,66E-01	0.000555832	23236	PLCB1
ENSG00000185924	3.228.426.275	251.843.444	0.460434127	546.969.543	4,51E-04	8,43E-01	146760	RTN4RL1
ENSG00000124762	7.141.275.838	2.565.310.721	0.65514296	3.915.650.292	9,02E+00	0.003684473	1026	CDKN1A
ENSG00000186891	2.966.447.378	2.681.094.337	0.458834824	5.843.266.887	5,12E-04	1,33E-01	8784	TNFRSF18
ENSG00000140968	2.311.676.926	2.743.008.148	0.657164244	417.400.699	2,99E+00	0.001576289	3394	IRF8
ENSG00000177494	1.255.729.114	2.757.344.589	0.531566811	5.187.202.305	2,13E-02	3,22E+00	79413	ZBED2
ENSG00000237161	2.947.970.606	3.090.042.505	0.79236061	3.899.793.185	9,63E+00	0.003869296	NA	
ENSG00000205683	8.958.078.382	3.176.896.343	0.516131112	6.155.211.864	7,50E-05	2,60E-02	8110	DPF3
ENSG00000204262	1.187.725.784	33.206.939	0.769396204	4.315.973.855	1,59E+00	0.00098647	1290	COL5A2
ENSG00000149294	1.150.135.743	3.633.956.534	0.774765294	4.690.396.641	2,73E-01	0.000247085	4684	NCAM1
ENSG00000250337	6.770.577.196	3.788.621.655	0.809515854	4.680.108.038	2,87E-01	0.000255062	643401	PURPL
ENSG00000140470	2.869.493.448	3.933.623.785	0.960186685	4.096.728.111	4,19E+00	0.002065283	170691	ADAMTS17
ENSG00000122877	3.722.295.878	3.971.870.171	0.884595521	4.490.041.018	7,12E-01	0.000524684	1959	EGR2
ENSG00000140836	2.520.332.334	400.856.733	0.883535247	4.536.963.684	5,71E-01	0.000442869	388289	ZFHX3
ENSG00000125740	1.269.098.997	4.075.113.713	0.736211657	5.535.247.475	3,11E-03	6,39E-01	2354	FOSB
ENSG00000266088	2.249.012.976	4.144.352.181	0.5686155	7.288.496.671	3,13E-08	3,52E-05	NA	
ENSG00000142185	1.615.944.961	4.558.814.951	0.664247935	6.863.122.502	6,74E-07	6,14E-04	7226	TRPM2
ENSG00000172348	6.026.782.947	4.628.129.249	0.68871297	6.719.968.192	1,82E-06	1,26E-03	10231	RCAN2
ENSG00000103056	956.294.226	5.929.014.601	0.729842481	8.123.690.733	4,52E-11	8,25E-08	55512	SMPD3

Supplementary Table 2.

TCR sequences of the single cell clones profiled in this study. The sequences were aligned and annotated using the IMGT V-QUEST tool[29, 30].

Clone ID	TCRγ	CDR3γ	CDR3γ length	TCRδ	CDR3δ	CDR3δ length
C1	TRGV9*01TRGJP*01	CALWEVRELGKKIKVF	14	TRDV2*02TRDD2*01TRDJ1*01	CACDP**V**PSIHDTDKLIF	15
C2/18	TRGV9*01TRGJP*01	CALWEVRELGKKIKVF	14	TRDV2*02TRDD3*01TRDJ1*01	CACDQ**A**GGPDKLIF	12
C3	TRGV9*01TRGJP*01	CALWEVGLGKKIKVF	13	TRDV2*02TRDD3*01TRDJ1*01	CACDT**V**SGGYQYTDKLIF	16
C4/14	TRGV9*01TRGJP*01	CALWEVSGELGKKIKVF	15	TRDV2*02TRDD2*01TRDD3*01TRDJ1*01	CACDT**L**ALGDTDKLIF	14
C5	TRGV9*01TRGJP*01	CALWEVQELGKKIKVF	14	TRDV2*02TRDD3*01TRDJ1*01	CACDL**L**APGDTSFTDKLIF	17
C6/9/13	TRGV9*01TRGJP*01	CALWEVKRELGKKIKVF	15	TRDV2*02TRDD3*01TRDJ1*01	CACDT**V**VLGTGGYRDDKLIF	18
C8	TRGV9*01TRGJP*01	CALWEATELGKKTKVF.	14	TRDV2*02TRDD2*01TRDD3*01TRDJ1*01	CACDR**L**PGLDTTDKLIF	15
C12	TRGV9*01TRGJP*01	CALWEVPQELGKKIKVF	15	TRDV2*02TRDD2*01TRDJ1*01	CACDT**V**SSPRGKLIF	13
C7/11/15	TRGV9*01TRGJP*01	CALWEVRELGKKIKVF	14	TRDV2*02TRDD3*01TRDJ3*01	CACDM**G**DASSWDTRQMFF	16
C17	n.a.	n.a.	n.a.	TRDV2*02TRDD3*01TRDJ1*01	CACDT**L**RQTGGPTDKLIF	16
D14	TRGV9*01TRGJP*01	CALWEVQEELGKKIKVF	15	TRDV2*03TRDD3*01TRDJ1*01	CACDA**L**LGDTTDKLIF	14
D84	TRGV9*01TRGJP*01	CALWEELGKKIKVF	12	TRDV2*03TRDD3*01TRDJ1*01	CACED**L**GAREVDKLIF	14
D113	TRGV9*01TRGJP*01	CALWGGELGKKIKVF	13	TRDV2*03TRDD1*01TRDJ1*01	CACDL**L**GDNTDKLIF	13
D138/ 325	TRGV9*01TRGJP*01	CALWVRELGKKIKVF	13	TRDV2*03TRDD3*01TRDJ1*01	CACDT**V**GDTDDKLIF	13
D145	TRGV9*01TRGJP*01	CALWEVHRELGKKIKVF	15	TRDV2*03TRDD3*01TRDJ1*01	CACDT**L**GVNTDKLIF	13
D158	TRGV9*01TRGJP*01	CALWEVRELGKKIKVF	14	TRDV2*03TRDD3*01TRDJ1*01	CACDP**L**LGDTGDKLIF	14
D170	TRGV9*01TRGJP*01	CALWAELGKKIKVF	12	TRDV2*03TRDD3*01TRDJ3*01	CACDT**L**GDYQARQMFF	14
D172	TRGV9*01TRGJP*01	CALWEVEPLGKKIKVF	14	TRDV2*03TRDD3*01TRDJ1*01	CACSV**L**GDTPTDKLIF	14
D181	TRGV9*01TRGJP*01	CALWEVNQELGKKIKVF	15	TRDV2*03TRDD3*01TRDJ1*01	CACDT**V**SWGTGFNTDKLIF	17
D233/269/327	TRGV9*01TRGJP*01	CALWEAQELGKKIKVF	14	TRDV2*03TRDD3*01TRDJ1*01	CACDP**L**GDREYTDKLIF	15
D258	TRGV9*01TRGJP*01	CALWEVQQELGKKIKVF	15	TRDV2*03TRDD3*01TRDJ1*01	CACDT**L**GARVTDKLIF	14
D289	TRGV9*01TRGJP*01	CALWEPGQELGKKIKVF	16	TRDV2*03TRDD3*01TRDJ1*01	CACDN**I**LGKNADKLIF	15
D306	TRGV9*01TRGJP*01	CALWEVLELGKKIKVF	14	TRDV2*03TRDD1*01TRDJ1*01	CACDV**L**GNIDKLIF	12

Supplementary references

1. June, C.H., et al., *CAR T cell immunotherapy for human cancer.* Science, 2018. **359**(6382): p. 1361–1365.
2. Cappell, K.M. and J.N. Kochenderfer, *Long-term outcomes following CAR T cell therapy: what we know so far.* Nat Rev Clin Oncol, 2023. **20**(6): p. 359–371.
3. Liu, Y., et al., *Optimizing the manufacturing and antitumour response of CAR T therapy.* Nature Reviews Bioengineering, 2023. **1**(4): p. 271–285.
4. Ma, C., et al., *Multifunctional T-cell analyses to study response and progression in adoptive cell transfer immunotherapy.* Cancer Discov, 2013. **3**(4): p. 418–29.
5. Bai, Z., et al., *Single-cell CAR T atlas reveals type 2 function in 8-year leukaemia remission.* Nature, 2024. **634**(8034): p. 702–711.
6. Laskowski, T.J., A. Biederstadt, and K. Rezvani, *Natural killer cells in antitumour adoptive cell immunotherapy.* Nat Rev Cancer, 2022. **22**(10): p. 557–575.
7. Marin, D., et al., *Safety, efficacy and determinants of response of allogeneic CD19-specific CAR-NK cells in CD19(+) B cell tumors: a phase 1/2 trial.* Nat Med, 2024. **30**(3): p. 772–784.
8. Beringer, D.X., et al., *Disrupting the balance between activating and inhibitory receptors of gammadeltaT cells for effective cancer immunotherapy.* Nat Rev Cancer, 2025. **25**(8): p. 590–612.
9. Willcox, B.E. and C.R. Willcox, *gammadelta TCR ligands: the quest to solve a 500-million-year-old mystery.* Nat Immunol, 2019. **20**(2): p. 121–128.
10. Gutierrez-Arcelus, M., et al., *Lymphocyte innateness defined by transcriptional states reflects a balance between proliferation and effector functions.* Nat Commun, 2019. **10**(1): p. 687.
11. Pizzolato, G., et al., *Single-cell RNA sequencing unveils the shared and the distinct cytotoxic hallmarks of human TCRVdelta1 and TCRVdelta2 gammadelta T lymphocytes.* Proc Natl Acad Sci U S A, 2019. **116**(24): p. 11906–11915.
12. Herrmann, T. and M.M. Karunakaran, *Phosphoantigen recognition by Vgamma9Vdelta2 T cells.* Eur J Immunol, 2024. **54**(11): p. e2451068.
13. McMurray, J.L., et al., *Transcriptional profiling of human Vδ1 T cells reveals a pathogen-driven adaptive differentiation program.* Cell Reports, 2022. **39**(8).
14. Di Lorenzo, B., et al., *Broad Cytotoxic Targeting of Acute Myeloid Leukemia by Polyclonal Delta One T Cells.* Cancer Immunol Res, 2019. **7**(4): p. 552–558.
15. Capsomidis, A., et al., *Chimeric Antigen Receptor-Engineered Human Gamma Delta T Cells: Enhanced Cytotoxicity with Retention of Cross Presentation.* Mol Ther, 2018. **26**(2): p. 354–365.
16. Deniger, D.C., J.S. Moyes, and L.J. Cooper, *Clinical applications of gamma delta T cells with multivalent immunity.* Front Immunol, 2014. **5**: p. 636.
17. Tan, L.K., et al., *A fetal wave of human type 3 effector γδ cells with restricted TCR diversity persists into adulthood.* Science Immunology, 2021. **6**(58).
18. Ryan, P.L., et al., *Heterogeneous yet stable Vdelta2(+) T-cell profiles define distinct cytotoxic effector potentials in healthy human individuals.* Proc Natl Acad Sci U S A, 2016. **113**(50): p. 14378–14383.
19. Ng, J.W.K., et al., *Cord blood-derived V(δ)2(+) and V(δ)2(-) T cells acquire differential cell state compositions upon in vitro expansion.* Sci Adv, 2023. **9**(24): p. eadf3120.
20. Martínez, D.S., et al., *Generation and proof-of-concept for allogeneic CD123 CAR-Delta One T (DOT) cells in acute myeloid leukemia.* Journal for Immunotherapy of Cancer, 2022. **10**(9).
21. Lee, D., et al., *Unlocking the potential of allogeneic Vδ2 T cells for ovarian cancer therapy through CD16 biomarker selection and CAR/IL-15 engineering.* Nature Communications, 2023. **14**(1): p. 6942.

22. Cazzetta, V., et al., *NKG2A expression identifies a subset of human Vδ2 T cells exerting the highest antitumor effector functions.* Cell Rep, 2021. **37**(3): p. 109871.
23. Marcu-Malina, V., et al., *Redirecting alphabeta T cells against cancer cells by transfer of a broadly tumor-reactive gammadeltaT-cell receptor.* Blood, 2011. **118**(1): p. 50–9.
24. Ribot, J.C., et al., *Human γδ Thymocytes Are Functionally Immature and Differentiate into Cytotoxic Type 1 Effector T Cells upon IL-2/IL-15 Signaling.* Journal of Immunology, 2014. **192**(5): p. 2237–2243.
25. Nicolasen, M.J.T., et al., *Autologous profiling reveals inter-patient heterogeneity in Vdelta2(+)gammadeltaTIL responses to glioblastoma driven by extracellular matrix-BTN3A axis.* J Immunother Cancer, 2025. **13**(12).
26. Vyborova, A., et al., *gamma9delta2T cell diversity and the receptor interface with tumor cells.* J Clin Invest, 2020. **130**(9): p. 4637–4651.
27. Shannon, P., et al., *Cytoscape: a software environment for integrated models of biomolecular interaction networks.* Genome Res, 2003. **13**(11): p. 2498–504.
28. Hänzelmann, S., R. Castelo, and J. Guinney, *GSVA: gene set variation analysis for microarray and RNA-Seq data.* Bmc Bioinformatics, 2013. **14**.
29. Giudicelli, V., X. Brochet, and M.P. Lefranc, *IMGT/V-QUEST: IMGT standardized analysis of the immunoglobulin (IG) and T cell receptor (TR) nucleotide sequences.* Cold Spring Harb Protoc, 2011. **2011**(6): p. 695–715.
30. Brochet, X., M.P. Lefranc, and V. Giudicelli, *IMGT/V-QUEST: the highly customized and integrated system for IG and TR standardized V-J and V-D-J sequence analysis.* Nucleic Acids Res, 2008. **36**(Web Server issue): p. W503–8.
31. Liberzon, A., et al., *The Molecular Signatures Database (MSigDB) hallmark gene set collection.* Cell Syst, 2015. **1**(6): p. 417–425.
32. Li, S., et al., *Molecular signatures of antibody responses derived from a systems biology study of five human vaccines.* Nat Immunol, 2014. **15**(2): p. 195–204.
33. Kiner, E., et al., *Gut CD4+ T cell phenotypes are a continuum molded by microbes, not by TH archetypes.* Nature Immunology, 2021. **22**(2): p. 216–228.
34. Rath, J.A., et al., *Single-cell transcriptomics identifies multiple pathways underlying antitumor function of TCR- and CD8alphabeta-engineered human CD4(+) T cells.* Sci Adv, 2020. **6**(27): p. eaaz7809.
35. Szabo, S.J., et al., *A novel transcription factor, T-bet, directs Th1 lineage commitment.* Cell, 2000. **100**(6): p. 655–69.
36. Pearce, E.L., et al., *Control of effector CD8+ T cell function by the transcription factor Eomesodermin.* Science, 2003. **302**(5647): p. 1041–3.
37. Bai, A., et al., *Kruppel-like factor 2 controls T cell trafficking by activating L-selectin (CD62L) and sphingosine-1-phosphate receptor 1 transcription.* J Immunol, 2007. **178**(12): p. 7632–9.
38. Hosken, N.A., et al., *The effect of antigen dose on CD4+ T helper cell phenotype development in a T cell receptor-alpha beta-transgenic model.* J Exp Med, 1995. **182**(5): p. 1579–84.
39. Tan, M.P., et al., *T cell receptor binding affinity governs the functional profile of cancer-specific CD8+ T cells.* Clin Exp Immunol, 2015. **180**(2): p. 255–70.
40. van Diest, E., et al., *Gamma delta TCR anti-CD3 bispecific molecules (GABs) as novel immunotherapeutic compounds.* J Immunother Cancer, 2021. **9**(11).
41. Sanchez Sanchez, G., et al., *Identification of distinct functional thymic programming of fetal and pediatric human γδ thymocytes via single-cell analysis.* Nature Communications, 2022. **13**(1): p. 5842.
42. Cramer, A., et al., *Early-life thymectomy leads to an increase of granzyme-producing gammadelta T cells in children with congenital heart disease.* Nat Commun, 2024. **15**(1): p. 9841.

43. Davey, M.S., et al., *The human Vdelta2(+) T-cell compartment comprises distinct innate-like Vgamma9(+) and adaptive Vgamma9(-) subsets.* Nat Commun, 2018. **9**(1): p. 1760.
44. Ravens, S., et al., *Human γδ T cells are quickly reconstituted after stem-cell transplantation and show adaptive clonal expansion in response to viral infection.* Nature Immunology, 2017. **18**(4): p. 393–+.
45. Grunder, C., et al., *gamma9 and delta2CDR3 domains regulate functional avidity of T cells harboring gamma9delta2TCRs.* Blood, 2012. **120**(26): p. 5153–62.
46. Starick, L., et al., *Butyrophilin 3A (BTN3A, CD277)-specific antibody 20.1 differentially activates Vgamma9Vdelta2 TCR clonotypes and interferes with phosphoantigen activation.* Eur J Immunol, 2017. **47**(6): p. 982–992.
47. Vyborova, A., et al., *γ9δ2 T-Cell Expansion and Phenotypic Profile Are Reflected in the CDR3δ Repertoire of Healthy Adults.* Frontiers in Immunology, 2022. **13**.
48. Dekkers, J.F., et al., *Uncovering the mode of action of engineered T cells in patient cancer organoids.* Nat Biotechnol, 2023. **41**(1): p. 60–69.
49. Hernandez-Lopez, P., et al., *Cis- and trans-binding chimeric costimulatory receptors enhance T-cell fitness and tumor control.* Cell Mol Immunol, 2026. **23**(1): p. 79–93.
50. Hernandez-Lopez, P., et al., *Dual targeting of cancer metabolome and stress antigens affects transcriptomic heterogeneity and efficacy of engineered T cells.* Nat Immunol, 2024. **25**(1): p. 88–101.
